# Machine learning prediction of self-rated health among adults under different physical exercise participation statuses

**DOI:** 10.3389/fpubh.2026.1882082

**Published:** 2026-10-09

**Authors:** Ruishi Fan, Xinyue Zhang, Jiayu Shen, Fu Sun

**Affiliations:** 1 College of Physical Education and Health, East China Normal University, Shanghai, China; 2 Key Laboratory of Adolescent Health Assessment and Exercise Intervention of Ministry of Education, East China Normal University, Shanghai, China; 3 China Basketball College, Beijing Sport University, Beijing, China

**Keywords:** adults, health prediction, machine learning, physical exercise participation, self-rated health

## Abstract

**Background:**

Self-rated health (SRH) reflects adults’ physical, psychological, and social well-being and is influenced by multiple determinants. Physical exercise is an important health-promoting behavior, yet evidence remains limited regarding the key predictors of SRH and their relative importance across different exercise participation groups.

**Objective:**

This study used machine learning to compare SRH prediction models across physical exercise participation, identify key predictors and feature-importance differences.

**Methods:**

Using data from the 2021 Chinese General Social Survey (CGSS2021), adults were divided into a Participate group and a Non-Participate group, and seven machine learning models, including Random Forests (RF), were developed to predict SRH status. Subsequently, the SHAP method was applied to quantify the contribution of individual features to model predictions.

**Results:**

Among the Participate group, the RF model exhibited a relatively high AUC on the test set; amongst Non-Participate group, the LGBM model exhibited a relatively high AUC on the test set, with the performance differences between the primary models in the two groups being generally small. ‘Health Impact’, ‘Age’ and ‘Depression’ were common core predictors across both groups, although their ranking of importance and distribution of contributions were not entirely consistent. Variables such as ‘Internet Use’ and ‘Social Friends’ also demonstrated some auxiliary predictive value. Further stratification by physical exercise frequency and sensitivity analyses revealed that, although there have been changes in model performance and the ranking of some features, the core predictive factors have remained broadly stable.

**Conclusion:**

The key predictors of SRH among adults with different levels of physical activity participation share commonalities but also exhibit certain differences. Predicting SRH in adults requires a comprehensive consideration of multi-level information, including functional status, demographic characteristics, recent emotional experiences and social behaviors. This study contributes to a further understanding of the relative contributions and heterogeneity of predictors of SRH among individuals with different levels of exercise participation and may provide a reference for future health research and stratified health promotion. Given that this study utilized cross-sectional survey data from a single year, the external validity and practical applicability of these models require further validation.

## 1 Introduction

Health status is a key indicator of individual quality of life, population well-being, and societal capacity for sustainable development, and it has long been a central focus of global public health research (1). In recent years, physical inactivity has increasingly been recognized as a major behavioral risk factor for public health (2). According to data from the World Health Organization, approximately 31% of adults worldwide did not meet the recommended levels of physical activity in 2022, corresponding to about 1.8 billion adults, and this proportion continues to increase (3). Previous studies have indicated that physical inactivity not only increases the risks of cardiovascular disease, type 2 diabetes, and depression but also exacerbates global medical and socioeconomic burdens (4). Existing evidence suggests that regular physical activity not only reduces the risk of all-cause mortality but also improves cardiopulmonary function, metabolic health, and mental well-being (5, 6). Furthermore, physical exercise has been shown to contribute to alleviating anxiety and depression, enhancing subjective well-being, and improving cognitive function (7, 8).

SRH (Self-Rated Health) is a comprehensive health measure widely adopted in public health research and provides a holistic assessment of multidimensional health information, including an individual’s physical functioning, psychological state and social adaptation (9). Although SRH is a subjective assessment of health, it not only provides a relatively accurate reflection of an individual’s current health status but also predicts chronic disease incidence, healthcare service utilization, functional limitations, and all-cause mortality risk (10, 11). The reliability and predictive validity of this comprehensive health outcome measure have been further validated in various countries and nationwide representative surveys, and it is widely applied in research on health behaviors, health equity and population health (12). Moreover, participation in physical exercise has been consistently associated with better SRH. Regular physical activity not only improves physical health but is also closely associated with higher levels of subjective well-being, quality of life and mental health (6, 7, 13). Furthermore, individuals with higher levels of physical activity are generally more likely to report better SRH (14). The health-promoting effects of physical activity may be mediated through several mechanisms, including improved sleep quality, reduced symptoms of anxiety and depression, and enhanced social adaptation (15, 16).

However, SRH is influenced by multiple dimensions of factors, including demographic characteristics, health behaviors, mental health and the social environment (17–19). Identifying the relative contributions of various factors to SRH is of great significance for understanding health inequalities and providing guidance for relevant health promotion research. Traditional statistical models are primarily used to estimate associations between variables and have certain limitations when dealing with high-dimensional predictors and complex data patterns (20, 21). In contrast, machine learning enables the identification of underlying patterns in high-dimensional data through a data-driven approach, whilst combining this with interpretability analysis to quantify the relative contribution of different predictors to the model’s output (20, 22). In recent years, interpretable machine learning has gradually been applied to health risk prediction and population health research (23–27). For example, de Souza-Lima et al. constructed an XGBoost model incorporating the SHAP explanation method based on nationally representative survey data to predict physical inactivity among adults and identified key influencing factors (27). Furthermore, interpretable machine learning has been widely applied to predicting a variety of health outcomes, providing novel insights into identifying health determinants (28, 29). More recently, its scope of application has further expanded into the field of population health; for instance, a multinational survey study utilized interpretable machine learning to predict children’s subjective well-being, finding that variables such as participation in physical activity, lifestyle and social support possessed high predictive importance (30).

In the field of SRH research, studies have begun to utilize machine learning to identify key predictors. For example, Clark et al. used large-scale survey data from the United States to predict SRH across different population groups and examined the predictive roles of demographic, socio-economic and health-related factors (25); Olsson et al. employed machine learning to analyze key determinants of SRH among older adults (31); Gumà-Lao and Arpino further examined the predictive relationships between health status, socio-economic factors and SRH, as well as differences across population subgroups (26). These studies indicate that machine learning is capable of identifying key predictors of SRH from multidimensional data; however, previous research has largely focused on specific age groups or specific populations, and physical activity is typically treated merely as one predictive variable within a model, with few studies constructing separate SRH prediction models based on physical activity participation status. Consequently, existing research still lacks a systematic comparison of whether different groups of physical activity participants share similar SRH predictive structures, and whether the same predictors make different relative contributions under different physical activity participation statuses. Treating physical activity participation status as a dimension for population stratification, and conducting predictive modeling and interpretability analyses separately within each group, will help to further examine the commonalities and heterogeneities of SRH influencing factors at the level of predictive structure. Furthermore, there remains a relative scarcity of interpretable machine learning studies based on national survey data from China that comprehensively examine multidimensional factors such as demographic characteristics, mental health, information usage and social behavior. Accordingly, this study utilizes data from the CGSS 2021 (Chinese General Social Survey 2021) to construct seven machine learning models, including RF, with the aim of: (1) Comparing the classification performance of different machine learning models in predicting SRH among adults across different levels of physical exercise participation; (2) Utilize SHAP to explain the key predictors of SRH and their relative contributions across different levels of physical activity participation; (3) Compare differences in the importance of key predictors across different levels of physical activity participation, to gain a deeper understanding of the commonalities and heterogeneities in the SRH prediction frameworks across different groups of physically active individuals, and to provide a reference for subsequent stratified health research and tailored health promotion.

## 2 Methods

### 2.1 Data and sample

This study employed a cross-sectional, secondary data analysis using data from the CGSS 2021 (32). The CGSS is one of the earliest nationwide, comprehensive and longitudinal social survey programs in China. Since 2003, it has continuously collected multidimensional information on residents’ demographic characteristics, socio-economic status, health, education and lifestyle, with the aim of providing high-quality data support for social science and public health research. CGSS 2021 surveyed a total of 8,148 adult respondents across 19 provinces (autonomous regions and municipalities) in China, including survey items relevant to this study such as health status, physical activity, mental health and social behavior. This study is reported in accordance with the TRIPOD+AI statement (33). In accordance with pre-defined inclusion criteria, respondents aged under 18 or over 80 were excluded. A complete-case analysis was then carried out on the sample comprising participants who met the age criteria (34) was conducted to exclude respondents with missing values for the outcome variable or any of the predictor variables, resulting in the final inclusion of 7,529 complete cases for model construction and analysis. The number and patterns of missing values for each variable, as well as a comparison of the baseline characteristics between those ultimately included in the analysis and those excluded due to missing data, are presented in Supplementary Table 5. The CGSS is the earliest nationally representative longitudinal survey project conducted by academic institutions in mainland China. The initial CGSS survey was carried out in accordance with the general ethical principles outlined in the Declaration of Helsinki. The Institutional Review Board of Renmin University of China oversaw research ethics. All participants signed an informed consent form prior to taking part in the survey. We have been authorized to use the publicly available CGSS data. As this study constitutes a secondary analysis of a database that contains no personally identifiable information and has been anonymised, and as there was no contact with participants, no additional ethical approval was required. The participant selection process is shown in Figure 1.

**Figure 1:**
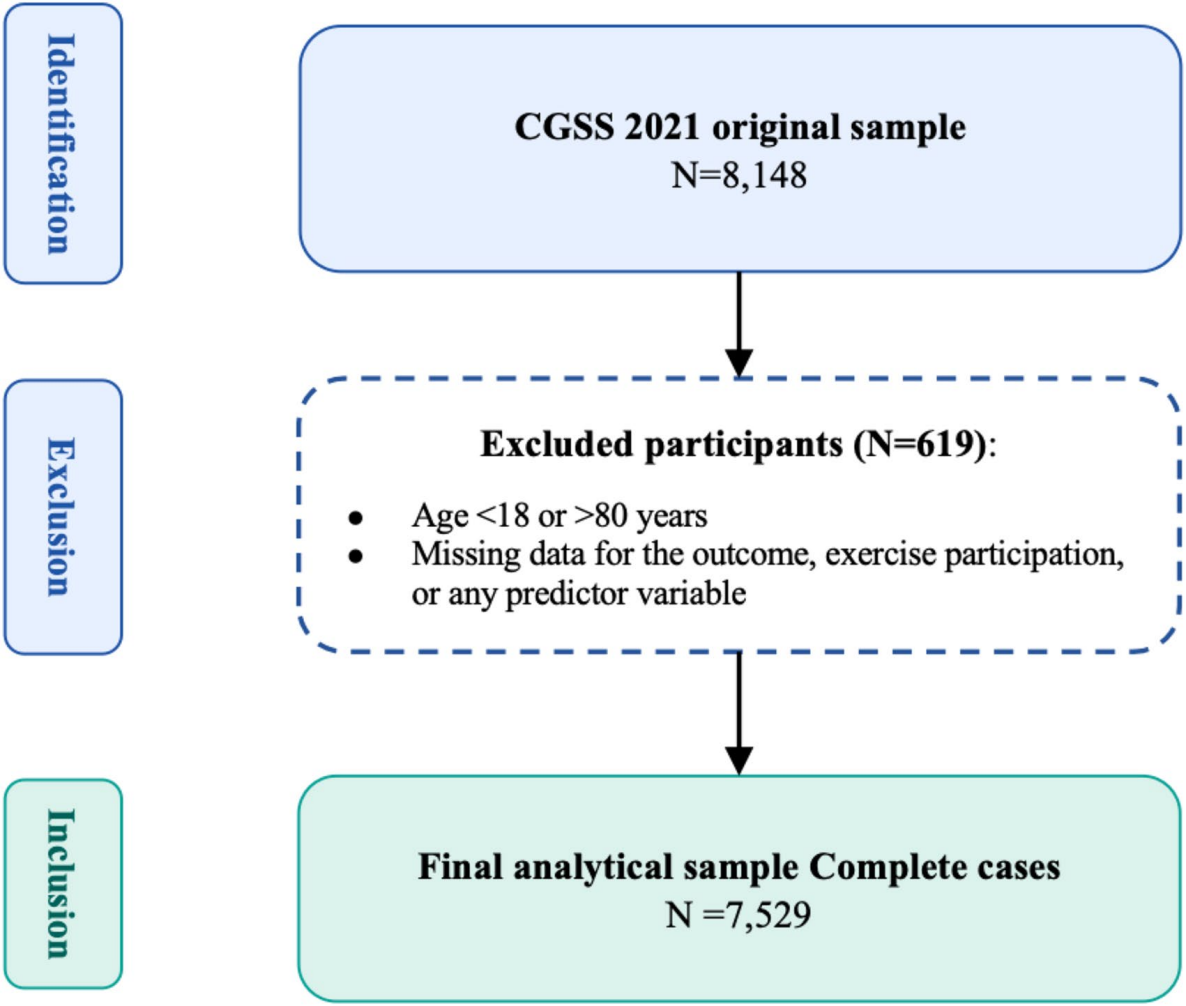
Flowchart of participant selection and sample inclusion.

### 2.2 Data collection

#### 2.2.1 Variables

In this study, participants assessed their own health status in response to the question ‘How would you rate your current physical health?’ in the CGSS questionnaire. This variable was measured on a five-point scale comprising very unhealthy, relatively unhealthy, fair, relatively healthy and very healthy. In this study, the primary analysis focused on identifying individuals with a lower SRH status; therefore, the original five-level ordinal variable was further categorized into a binary outcome with clear distinctions between health statuses. Previous studies have also shown that SRH is frequently used to construct classification indicators for higher and lower health statuses (25, 26, 31). There is a consistent gradient relationship between SRH and subsequent adverse health outcomes; as SRH levels decline, the risk of mortality gradually increases. Compared with the highest level of SRH, the risk of all-cause mortality is already significantly elevated among those rated as fair, and continues to rise as SRH deteriorates further. Studies have shown that, compared with an SRH level of at least good, a fair rating is associated with a higher risk of all-cause mortality (35, 36). Similar findings have been observed in other studies (37–39). This evidence suggests that fair is not merely a neutral health status, but is closer to lower levels of SRH on the health risk continuum. Consequently, this study defines fairly healthy and very healthy as the Healthy group, and fair, fairly unhealthy and very unhealthy as the Unhealthy group. To further assess the consistency of the predictive results under different outcome definitions, this study also retained the original five-category SRH classification for sensitivity analysis.

The physical activity variable was used to measure respondents’ participation in physical activity. The World Health Organization’s 2020 Physical Activity Guidelines state that replacing sedentary behavior with physical activity of any intensity (including light-intensity activity) yields health benefits, and emphasize that engaging in some level of physical activity is preferable to being completely inactive (2). Previous studies have further demonstrated that, compared with complete inactivity, lower levels of physical activity are associated with a reduced risk of all-cause mortality and cardiovascular disease (40, 41). The physical exercise variable was used to measure respondents’ participation in physical activity. Based on responses regarding the frequency of physical exercise in the questionnaire, this study defined respondents who answered daily, several times a week, several times a month and several times a year or less as the Participate group, and those who answered never as the Non-Participate group, in order to distinguish between those who engage in physical exercise and those who do not engage in it at all.

#### 2.2.2 Control variables

Based on previous research frameworks on health behaviors and SRH (42, 43), this study categorized the variables into four dimensions for analysis. Demographic characteristics included age, gender, ethnicity, body mass index (BMI), and educational attainment. Age referred to chronological age; gender was categorized as male or female; ethnicity was classified as Han Chinese or other ethnic minorities; BMI was calculated using the formula BMI = weight (kg)/height (m)^2^; and educational attainment was categorized as illiterate/elementary school or below, junior high school, senior high school, college or above, and graduate school or above. Health and psychological status included the frequency of health problems affecting daily activities and the frequency of depressive symptoms over the past four weeks. The information usage dimension includes frequency of internet use, whether the respondent owns a personal mobile phone, and whether they have accessed the internet in the past six months. The social behavior dimension includes participation in social activities, the frequency of social and leisure activities with neighbors and friends, and levels of social trust. For variable definitions and coding methods (see Supplementary Table 3).

### 2.3 Data preprocessing and feature selection

Data preprocessing is a critical step in machine learning modeling, as it improves data quality, ensures data consistency, and enhances the stability of model training and the reliability of prediction results (44). In this study, the raw data were first subjected to necessary cleaning,using complete-case analysis, samples with missing values for the dependent variable or any of the included variables were excluded; outliers were checked; and the correct variable types were ensured (34). This study did not employ one-hot encoding in order to preserve the original ordinal information of the variables.

The processed dataset was divided into participation and Non-Participate groups according to physical exercise participation. Within each group, the data were further split into training and testing sets at a 7:3 ratio, and stratified sampling was employed to ensure a consistent distribution of self-rated health status across the two sets. To enhance model stability and generalizability, 10-fold cross-validation combined with hyperparameter optimization was employed for model training. Cross-validation makes full use of the training data and reduces random bias arising from a single data split through repeated training and validation on different data subsets, thereby improving model stability and predictive performance. Furthermore, during model training, grid search was used to optimize model parameters, identify the optimal parameter combination, and reduce the risk of overfitting, set a fixed random seed (random seed = 42). Apart from the selection of complete cases and basic data cleaning, all data pre-processing, feature engineering, 10-fold stratified cross-validation and hyperparameter selection used for model development were carried out exclusively on the training set; the test set remained entirely separate and was not utilized during the model development and hyperparameter selection processes, and was only used for the final performance evaluation once the models had been trained and the hyperparameters finalized.

### 2.4 Machine learning models

This study developed seven machine learning models to predict SRH among adults with different levels of physical activity participation. It compared the predictive performance of these models across different physical activity groups and identified the key features associated with adults’SRH and differences in their relative importance were identified. The selected models included RF (45), decision tree (DT) (46), XGBoost (XGB) (47), LightGBM (LGBM) (48), support vector machine (SVM) (49), logistic regression (LR) (50) and k-nearest neighbors (KNN) (51). During the model training process, the training set was used for model fitting, whilst the test set was used to evaluate model performance. Model training, validation and hyperparameter optimization were all carried out using the aforementioned strategies to obtain the optimal model configurations for the ‘Participate’ and ‘Non-Participate’ groups (Table 1).

**Table 1:** Optimal hyperparameter settings for machine learning models in the Participate group and Non-Participate group.

Group	Model	Optimal hyperparameters
Participate	RF	max_depth = 7; min_samples_leaf = 10; n_estimators = 100
	DT	max_depth = 5; min_samples_leaf = 10
	XGB	colsample_bytree = 0.8; learning_rate = 0.05; max_depth = 3; n_estimators = 100; reg_alpha = 1; reg_lambda = 1; subsample = 0.8
	LGBM	colsample_bytree = 0.8; learning_rate = 0.05; max_depth = 3; num_leaves = 15; reg_alpha = 0.5; reg_lambda = 0.5; subsample = 0.8
	SVM	C = 0.1; kernel = rbf
	LR	C = 1
	KNN	n_neighbors = 7
Non Participate	RF	max_depth = 5; min_samples_leaf = 10; n_estimators = 100
	DT	max_depth = 3; min_samples_leaf = 5
	XGB	colsample_bytree = 0.8; learning_rate = 0.05; max_depth = 3; n_estimators = 100; reg_alpha = 1; reg_lambda = 1; subsample = 0.8
	LGBM	colsample_bytree = 0.8; learning_rate = 0.05; max_depth = 3; num_leaves = 15; reg_alpha = 0.5; reg_lambda = 0.5; subsample = 0.8
	SVM	C = 1; kernel = rbf
	LR	C = 0.1
	KNN	n_neighbors = 7

In order to comprehensively evaluate the predictive performance of the machine learning models developed, this study utilized accuracy, balanced accuracy, macro F1, ROC AUC, MCC, sensitivity and specificity as discriminative performance metrics, and the Brier score, calibration intercept and calibration slope as calibration performance metrics.

Furthermore, this study employed the area under the receiver operating characteristic curve (ROC-AUC) to evaluate the models’ classification performance. The ROC curve reflects the model’s ability to distinguish between positive and negative samples at different classification thresholds, whilst the AUC value is used to measure the model’s overall discriminatory performance. Compared with a single accuracy metric, ROC-AUC provides a comprehensive evaluation of the model’s discriminatory ability at different classification thresholds, thereby reducing the impact of threshold selection on the model evaluation results (52). Furthermore, this study employs a calibration curve to assess the consistency between the model’s predicted probabilities and actual observed outcomes, and combines the Brier score, calibration intercept and calibration slope to evaluate the model’s calibration performance, thereby comprehensively assessing the model’s predictive performance. To enhance the model’s interpretability, this study utilizes the SHapley Additive Explanations (SHAP) method to conduct interpretative analysis of the model. SHAP is based on the theory of Shapley values in game theory; it quantifies the marginal contribution of each feature to the model’s predictive results to assess the importance of variables and the direction of their influence (53).

### 2.5 Additional analyses

To assess the robustness of the main findings, this study conducted a series of additional analyses (Supplementary material). All models were re-estimated using the original five-category SRH classification to evaluate the impact of the outcome variable coding on model performance and variable importance. Secondly, ‘daily’ and ‘several times a week’ were defined as the ‘regular participation’ group, whilst ‘several times a month’ and ‘several times a year or less’ were defined as the ‘occasional participation’ group, in order to assess the impact of different categorizations of physical exercise on the study results. Finally, given the potential conceptual overlap between the ‘Health Impact’ variable and the SRH outcome, an ablation analysis was conducted by rebuilding the models after excluding the ‘Health Impact’ variable (54) to assess its impact on model performance and feature importance.

### 2.6 Statistical analysis

Descriptive statistical analyses were performed using SPSS 27.0 software. Continuous variables are presented as mean and standard deviation (Mean (SD)), and differences between groups were assessed using t-tests; categorical variables are presented as frequency and percentage (*n* (%)), and comparisons between groups were assessed using the chi-squared test. This study focuses primarily on the individual-level predictive performance of SRH across different levels of physical activity participation, as well as the relative contributions of predictive factors; consequently, both the statistical analysis and the development of machine learning models were based on the actual analysis sample, without incorporating the CGSS survey’s sampling weights. A *p*-value of < 0.05 was considered statistically significant. The construction, training and performance evaluation of the machine learning models were carried out using Python version 3.13. Python libraries such as NumPy, Pandas, Scikit-learn, XGBoost, LightGBM, SHAP and Matplotlib were primarily used for data processing, model construction, performance evaluation and result visualization.

## 3 Results

### 3.1 Descriptive statistics

Statistical data show that this study included a total of 7,529 participants, of whom 5,025 engaged in physical exercise (66.7%) and 2,504 did not (33.3%). In the Participate group, there were 2,363 men (47.0%) and 2,662 women (53.0%), with a mean age of 47.35 (17.02); in the Non-Participate group, there were 1,046 men (41.8%) and 1,458 women (58.2%), with a mean age of 56.03 (14.42). Within the Participate group, 3,024 participants (60.2%) were in the healthy subgroup and 2,001 (39.8%) were in the non-healthy subgroup; in the Non-Participate group 1,102 participants (44.0%) were in the healthy subgroup and 1,402 (56.0%) in the non-healthy subgroup. Statistical analysis revealed significant correlations between participation in physical exercise and multiple health, psychological and socio-behavioral indicators (see Table 2); participation in physical exercise was closely associated with higher SRH levels and more favorable psychological and socio-behavioral characteristics.

**Table 2:** Descriptive characteristics of participants by physical exercise participation status.

Feature variable	Total (%) (*N* = 7,529)	Participate group (%) (*N* = 5,025, 66.7%)	Non-Participate group (%) (*N* = 2,504, 33.3%)	χ^2^/t	*p*
Age	50.24 (16.71)	47.35 (17.02)	56.03 (14.42)	23.166	<0.001
BMI	23.09 (3.69)	23.04 (3.59)	23.17 (3.88)	1.367	0.172
Health impact	3.98 (1.21)	4.14 (1.08)	3.66 (1.38)	15.232	<0.001
Depression	3.95 (1.08)	4.03 (1.01)	3.81 (1.19)	8.059	<0.001
Internet use	3.43 (1.64)	3.77 (1.50)	2.76 (1.69)	25.363	<0.001
Social trust	3.63 (1.00)	3.63 (0.96)	3.61 (1.06)	0.899	0.369
Neighbor social	4.27 (2.22)	4.26 (2.18)	4.28 (2.29)	0.298	0.765
Friend social	4.17 (1.95)	3.83 (1.82)	4.86 (2.03)	21.467	<0.001
Gender				18.603	<0.001
Female	4,120 (54.7)	2,662 (53.0)	1,458 (58.2)		
Male	3,409 (45.3)	2,363 (47.0)	1,046 (41.8)		
Ethnicity				5.075	0.024
Other ethnic minorities	556 (7.4)	347 (6.9)	209 (8.3)		
Han Chinese	6,973 (92.6)	4,678 (93.1)	2,295 (91.7)		
Education				1096.93	<0.001
Illiterate/primary school or below	2,298 (30.5)	1,035 (20.6)	1,263 (50.4)		
Junior high school	2,197 (29.2)	1,367 (27.2)	830 (33.1)		
Senior high/technical secondary/vocational school or equivalent	1,414 (18.8)	1,125 (22.4)	289 (11.5)		
College or above	1,506 (20.0)	1,387 (27.6)	119 (4.8)		
Postgraduate or above	114 (1.5)	111 (2.2)	3 (0.1)		
Phone				85.127	<0.001
Yes	7,228 (96.0)	4,898 (97.5)	2,330 (93.1)		
No	301 (4.0)	127 (2.5)	174 (6.9)		
Online recent				539.9	<0.001
Yes	5,473 (72.7)	4,076 (81.1)	1,397 (55.8)		
No	2056 (27.3)	949 (18.9)	1,107 (44.2)		
Social participation				180.506	<0.001
Participated	6,394 (84.9)	4,464 (88.8)	1930 (77.1)		
Did not participate	1,135 (15.1)	561 (11.2)	574 (22.9)		
SRH				176.405	<0.001
Unhealthy group (fair, relatively unhealthy, or very unhealthy)	3,403 (45.2)	2001 (39.8)	1,402 (56.0)		
Healthy group (relatively healthy or very healthy)	4,126 (54.8)	3,024 (60.2)	1,102 (44.0)		

Continuous variables are presented as mean (standard deviation, SD), and categorical variables are presented as *n* (%). Percentages in subgroup columns are calculated within each subgroup. For the χ^2^/t statistic column, *t* tests were used for continuous variables and χ^2^ tests were used for categorical variables. *p* values less than 0.001 are reported as <0.001.

### 3.2 Model performance metrics

In the ‘Participate’ group, the differences in ROC AUC across the models on the test set were relatively small, with RF exhibited a relatively high ROC AUC (0.808) (0.786–0.832), followed by XGBoost and LGBM at 0.807 (0.785–0.831) and 0.805 (0.782–0.829) respectively. When considering metrics such as accuracy, RF demonstrated relatively better overall predictive performance, whilst KNN’s overall performance was relatively weaker (Table 3).

**Table 3:** Performance of machine learning models for SRH prediction in the Participate group.

Group	Model	Accuracy 95% CI	Balanced accuracy 95% CI	Macro F1 95% CI	ROC AUC 95% CI	MCC 95% CI	Sensitivity 95% CI	Specificity 95% CI	Brier score 95% CI	Calibration intercept	Calibration slope
Participate	RF	0.752 (0.731–0.772)	0.717 (0.693–0.739)	0.724 (0.699–0.748)	0.808 (0.786–0.832)	0.471 (0.423–0.516)	0.888 (0.868–0.907)	0.547 (0.503–0.587)	0.171 (0.162–0.180)	−0.104	1.350
	DT	0.731 (0.712–0.751)	0.687 (0.665–0.710)	0.692 (0.668–0.717)	0.787 (0.764–0.810)	0.426 (0.381–0.472)	0.904 (0.885–0.922)	0.470 (0.430–0.508)	0.178 (0.168–0.188)	0.134	0.823
	XGB	0.752 (0.730–0.773)	0.723 (0.698–0.746)	0.729 (0.704–0.753)	0.807 (0.785–0.831)	0.471 (0.423–0.518)	0.867 (0.845–0.889)	0.578 (0.535–0.618)	0.169 (0.160–0.179)	−0.006	1.140
	LGBM	0.745 (0.723–0.765)	0.716 (0.693–0.739)	0.722 (0.698–0.746)	0.805 (0.782–0.829)	0.455 (0.407–0.500)	0.855 (0.831–0.877)	0.578 (0.535–0.618)	0.171 (0.161–0.180)	0.011	1.129
	SVM	0.734 (0.713–0.755)	0.696 (0.672–0.720)	0.702 (0.676–0.727)	0.792 (0.769–0.817)	0.430 (0.382–0.480)	0.882 (0.861–0.903)	0.510 (0.468–0.550)	0.177 (0.166–0.187)	0.030	1.044
	LR	0.741 (0.718–0.763)	0.717 (0.693–0.741)	0.722 (0.697–0.746)	0.801 (0.778–0.825)	0.449 (0.399–0.496)	0.833 (0.808–0.857)	0.602 (0.560–0.643)	0.173 (0.163–0.183)	0.049	1.048
	KNN	0.704 (0.682–0.726)	0.670 (0.646–0.695)	0.674 (0.649–0.700)	0.744 (0.718–0.770)	0.363 (0.313–0.414)	0.833 (0.808–0.856)	0.508 (0.467–0.548)	0.199 (0.187–0.210)	0.189	0.166

In the Non-Participate group, the differences in ROC AUC on the test set were relatively small across the various models, with LGBM exhibited a relatively high ROC AUC 0.806 (0.775–0.835), followed by SVM at 0.805 (0.773–0.834), and RF, XGBoost and LR all at 0.804. When considered in conjunction with other metrics, LGBM demonstrated relatively good overall predictive performance, whilst DT and KNN showed relatively weaker overall performance (Table 4).

**Table 4:** Performance of machine learning models for SRH prediction in the Non-Participate group.

Group	Model	Accuracy 95% CI	Balanced accuracy 95% CI	Macro F1 95% CI	ROC AUC 95% CI	MCC 95% CI	Sensitivity 95% CI	Specificity 95% CI	Brier Score 95% CI	Calibration intercept	Calibration slope
Non Participate	RF	0.742 (0.711–0.771)	0.746 (0.715–0.776)	0.741 (0.710–0.771)	0.804 (0.771–0.833)	0.488 (0.427–0.547)	0.776 (0.731–0.819)	0.715 (0.672–0.755)	0.176 (0.163–0.190)	−0.007	1.163
	DT	0.718 (0.685–0.749)	0.716 (0.682–0.748)	0.715 (0.682–0.746)	0.780 (0.747–0.811)	0.431 (0.365–0.493)	0.701 (0.650–0.749)	0.732 (0.689–0.772)	0.185 (0.169–0.202)	−0.027	0.746
	XGB	0.754 (0.722–0.783)	0.760 (0.729–0.789)	0.754 (0.722–0.782)	0.804 (0.772–0.833)	0.517 (0.455–0.575)	0.810 (0.767–0.852)	0.710 (0.667–0.751)	0.175 (0.160–0.192)	−0.017	0.831
	LGBM	0.754 (0.725–0.783)	0.760 (0.731–0.788)	0.754 (0.724–0.782)	0.806 (0.775–0.835)	0.516 (0.459–0.573)	0.807 (0.764–0.849)	0.713 (0.670–0.753)	0.175 (0.160–0.191)	−0.006	0.820
	SVM	0.749 (0.715–0.778)	0.750 (0.718–0.780)	0.747 (0.714–0.777)	0.805 (0.773–0.834)	0.497 (0.434–0.557)	0.764 (0.719–0.810)	0.736 (0.694–0.777)	0.178 (0.163–0.195)	−0.043	0.854
	LR	0.746 (0.714–0.775)	0.747 (0.715–0.777)	0.744 (0.712–0.774)	0.804 (0.770–0.833)	0.490 (0.427–0.551)	0.752 (0.704–0.798)	0.741 (0.701–0.781)	0.178 (0.162–0.195)	−0.016	0.799
	KNN	0.731 (0.699–0.762)	0.733 (0.700–0.764)	0.730 (0.698–0.760)	0.783 (0.749–0.813)	0.462 (0.398–0.524)	0.743 (0.695–0.792)	0.722 (0.677–0.762)	0.191 (0.175–0.209)	−0.108	0.190

Figure 2 shows the ROC curves for the seven machine learning models in the ‘Participate’ group. The curves for all models lie above the random classification baseline. The RF model exhibits a larger area under the curve, followed by XGBoost and LightGBM, which are relatively close to one another, whilst the KNN model has the smallest area under the curve. Overall, the RF model demonstrates relatively good discriminatory power in the ‘Participate’ group.

**Figure 2:**
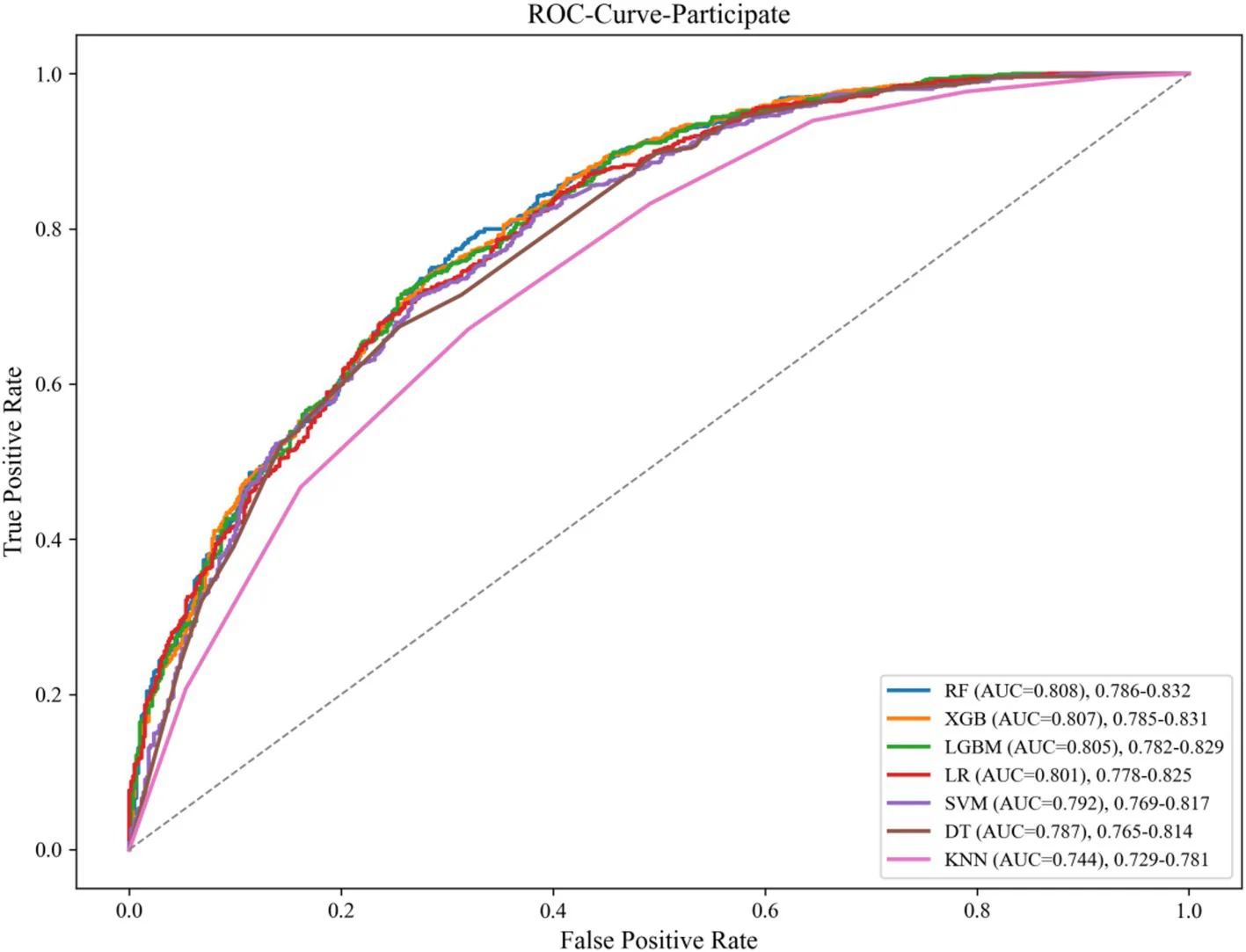
ROC curves of machine learning models in the Participate group.

Figure 3 shows the ROC curves for the seven machine learning models in the Non-Participate group. The curves for all models lie above the random classification baseline. The LGBM model exhibits a larger area under the curve (AUC), with the AUCs for SVM, XGBoost, RF and LR being relatively close to that of the LGBM model, whilst the DT model has the smallest AUC. Overall, the differences between the main models are minor, with the LGBM model demonstrating relatively good discriminatory performance in the Non-Participate group.

**Figure 3:**
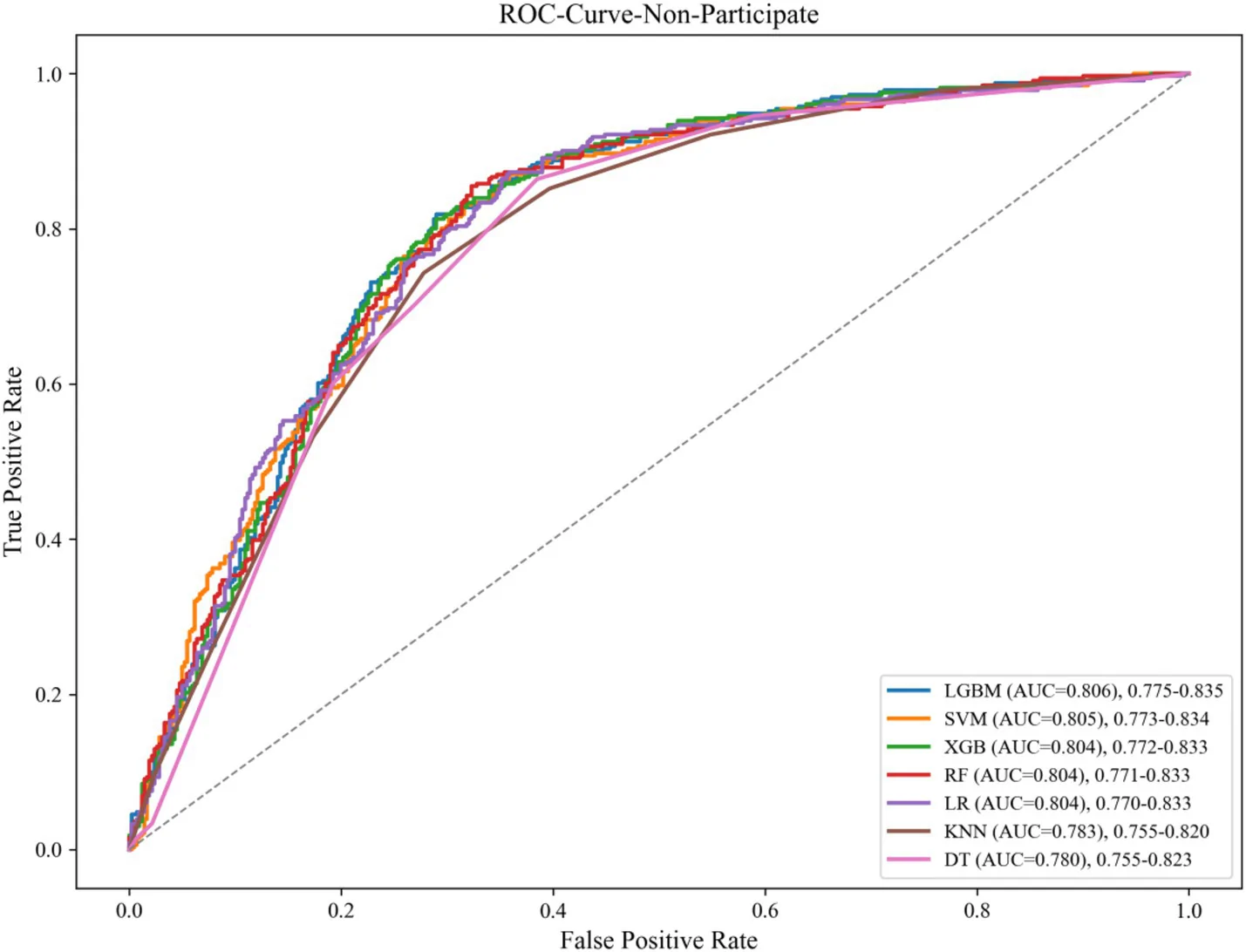
ROC curves of machine learning models in the Non-Participate group.

### 3.3 Model calibration

Figure 4 shows the calibration curves for the seven machine learning models in the ‘Participate’ group. Overall, the predicted probabilities of each model show good agreement with the actual observed results, and the calibration curves generally lie close to the ideal diagonal. Among these, the calibration curves for the XGBoost, LGBM and RF models align closely with the ideal diagonal; by contrast, the KNN model deviates more noticeably from the ideal diagonal in the high predicted probability range. Overall, all models demonstrate good calibration performance, with the XGBoost model particularly well.

**Figure 4:**
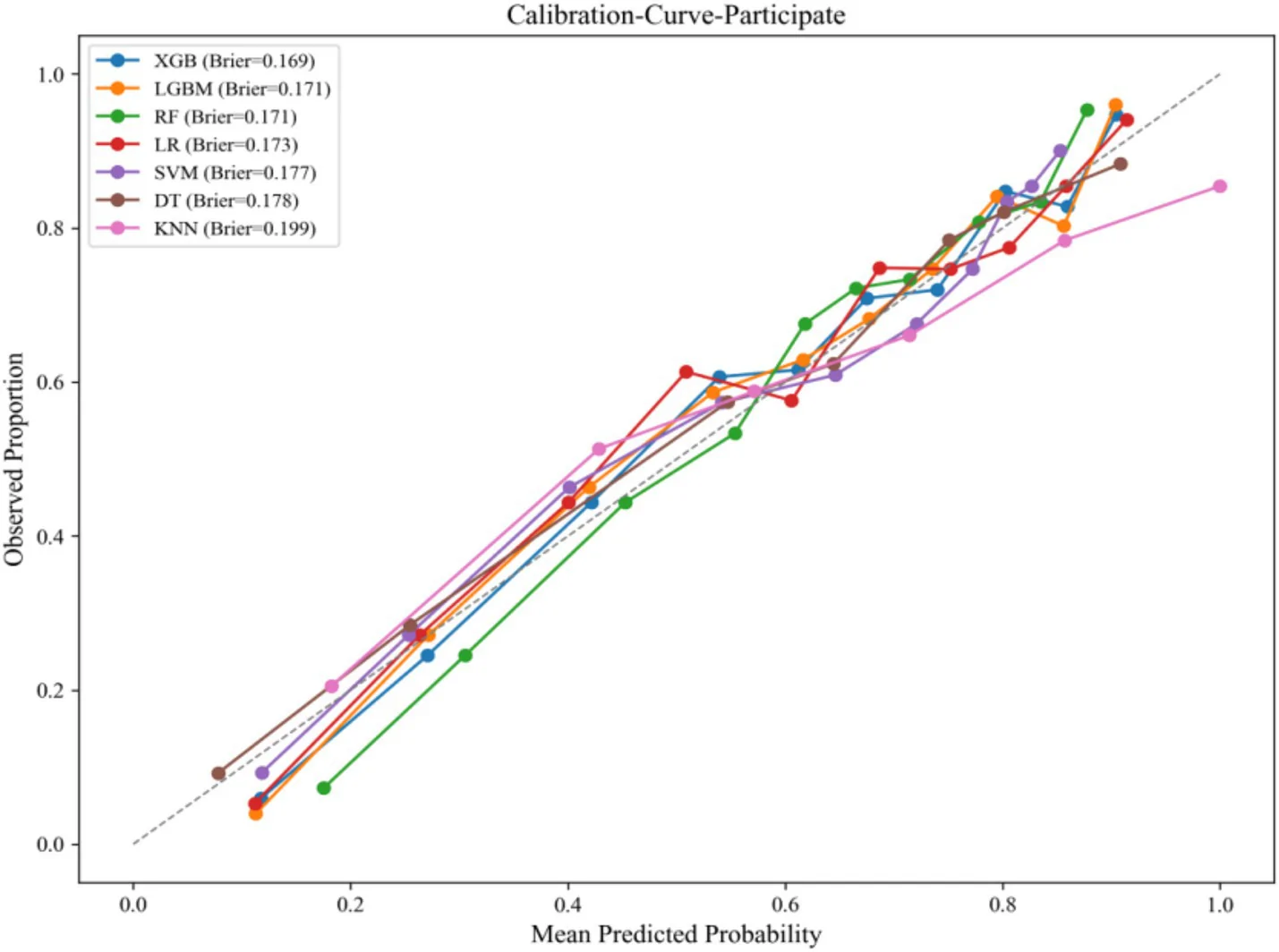
Calibration curves of machine learning models in the Participate group.

Figure 5 shows the calibration curves for the seven machine learning models in the Non-Participate group. Overall, the calibration curves for all models align well with the ideal diagonal. Among these, the calibration curves for the LGBM, XGBoost and RF models are generally closer to the ideal diagonal, whilst the DT and KNN models deviate relatively significantly from the ideal diagonal in certain probability intervals. Overall, all models demonstrated good calibration performance, with the LGBM and XGBoost models particularly well.

**Figure 5:**
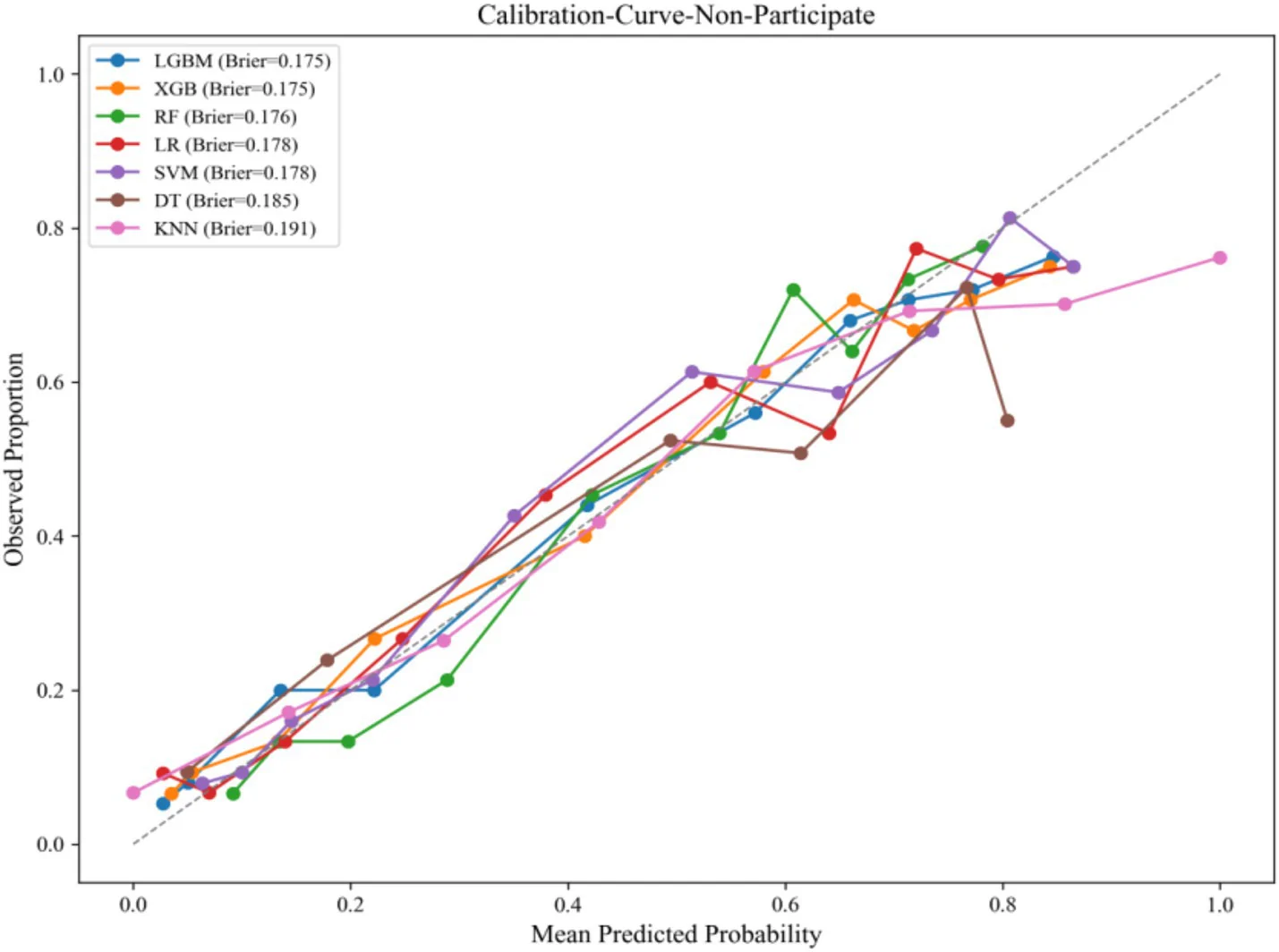
Calibration curves of machine learning models in the Non-Participate group.

### 3.4 Model interpretability

To further explain the model’s predictive results and the contribution of each feature to the model’s output, this study selected the RF model with a relatively high ROC-AUC value from the Participate group and the LGBM model with a relatively high ROC-AUC value from the Non-Participate group, and conducted an interpretability analysis using the SHAP method. Based on game theory, the SHAP method decomposes the model’s prediction results into the marginal contributions of individual features, thereby quantitatively assessing the direction and strength of the influence of different features on the prediction results. Positive SHA*p* values indicate that a feature promotes the model’s output, while negative SHAP values indicate that it inhibits the prediction results. Through SHAP summary scatter plots, average SHAP importance plots, and individual explanation plots, the importance and direction of each feature’s influence on the model’s prediction results can be intuitively demonstrated at both the overall and individual levels.

In the SHAP summary scatter plot for the Participate group (Figure 6), there are significant differences in the direction and magnitude of contributions from different features to the model output. Colors ranging from blue to red represent feature values from low to high, respectively, while the SHAP values on the x-axis indicate the direction and magnitude of a feature’s influence on the prediction result: positive SHAP values indicate a promoting effect on the model’s prediction of a healthy state, whereas negative SHAP values indicate a reduction in the probability of a healthy prediction. The results show that Health Impact has the greatest influence on the model output; high Health Impact values are primarily distributed in the positive SHAP region, while low values are more frequently found in the negative SHAP region. Age exhibits the opposite trend, with higher age values corresponding more often to negative SHAP values, suggesting that increasing age is associated with a lower probability of a healthy prediction. Depression also makes a significant contribution. Additionally, higher values for Internet Use and Education are generally more prevalent in regions with positive SHAP values, while the SHAP values for variables such as Gender, Ethnicity, and Social Participation are mostly close to 0, indicating a relatively limited impact on the model’s output.

**Figure 6:**
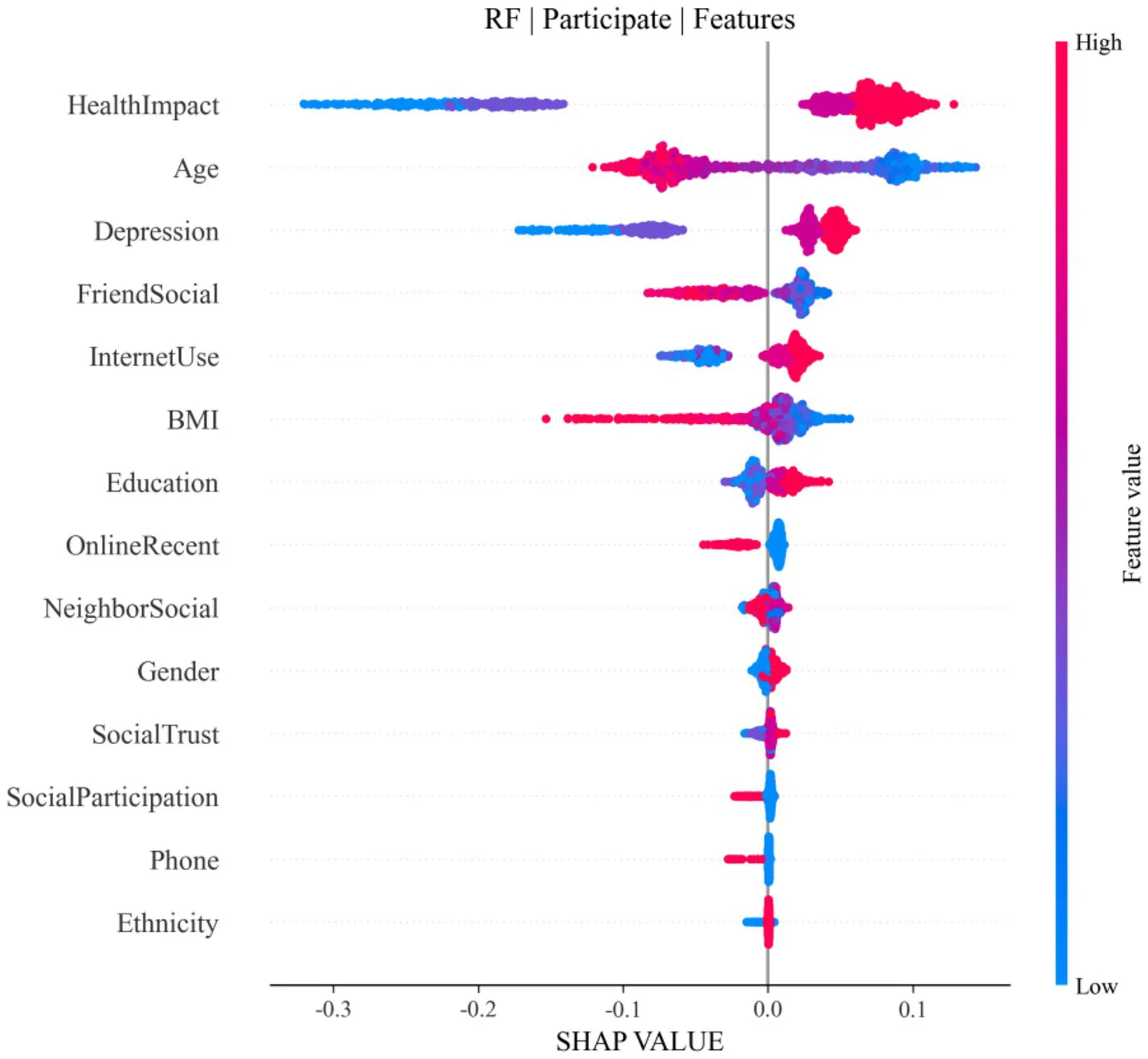
SHAP summary plot of feature contributions in the Participate group.

Figure 7 shows the ranking of average absolute SHAP values for each feature in the Participate group. Health Impact is the most important predictor, followed by Age and Depression, the contributions of these three are significantly higher than those of other features, indicating that perceived health status, age, and mental health are the core factors influencing the SRH predictions of individuals who participate in physical exercise. Friend Social, Internet Use and BMI fall into the second tier in terms of importance, whilst factors such as Social Participation are of lesser importance.

**Figure 7:**
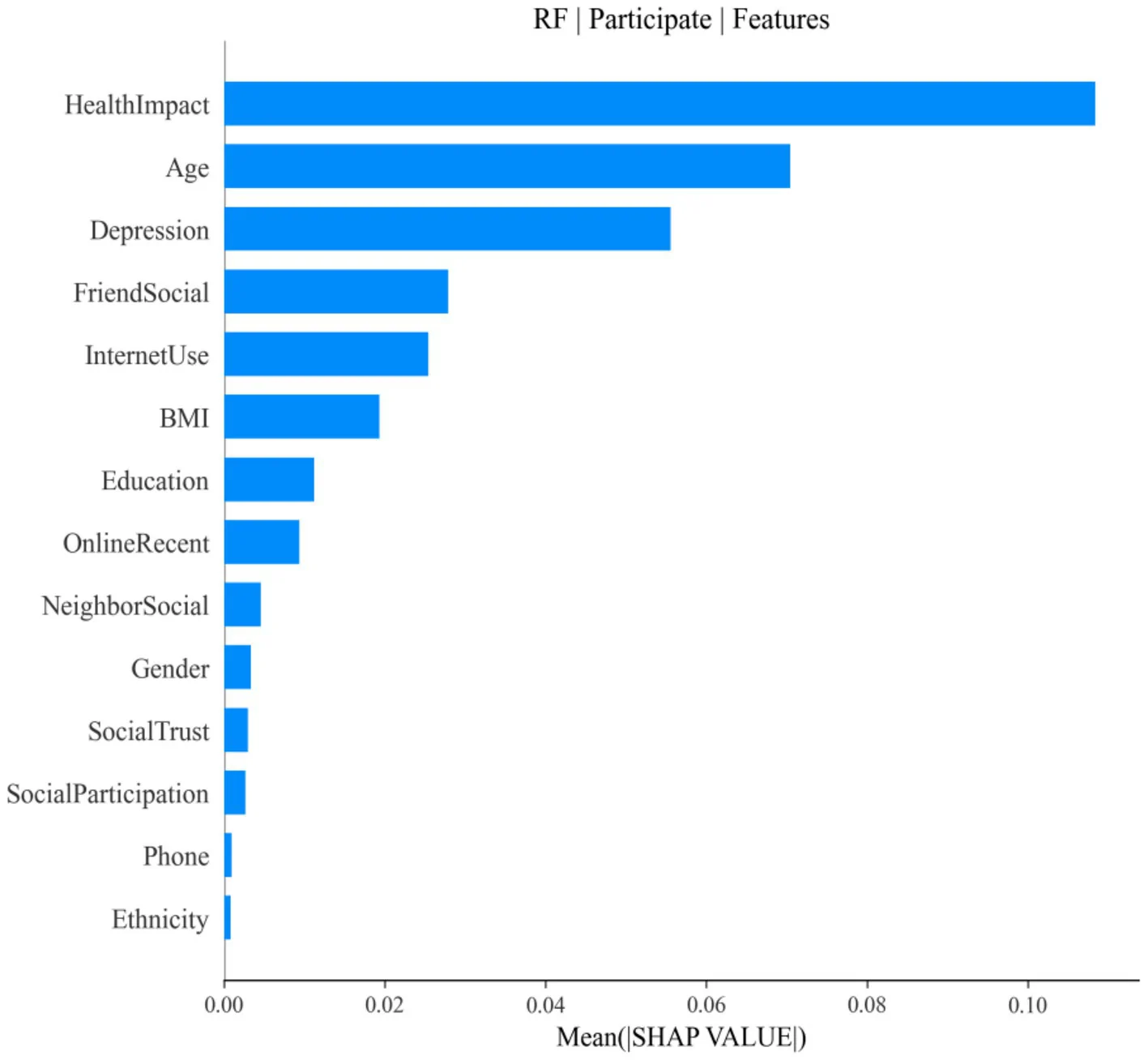
Mean absolute SHAP value importance ranking in the Participate group.

Figure 8 displays the SHAP individual feature contribution explanation plot for a single sample in the Participate group. The SHAP model output for this sample is 0.077, which is significantly lower than the model’s baseline value of E(f(X)) = 0.602. Among these, ‘Health Impact’ makes the largest negative contribution to the prediction result, followed by ‘Depression’, ‘Age’, ‘Internet Use’ and ‘BMI’; these variables collectively reduce the probability of a healthy outcome for this sample. ‘Online Recent’ also exerts a certain negative influence.

**Figure 8:**
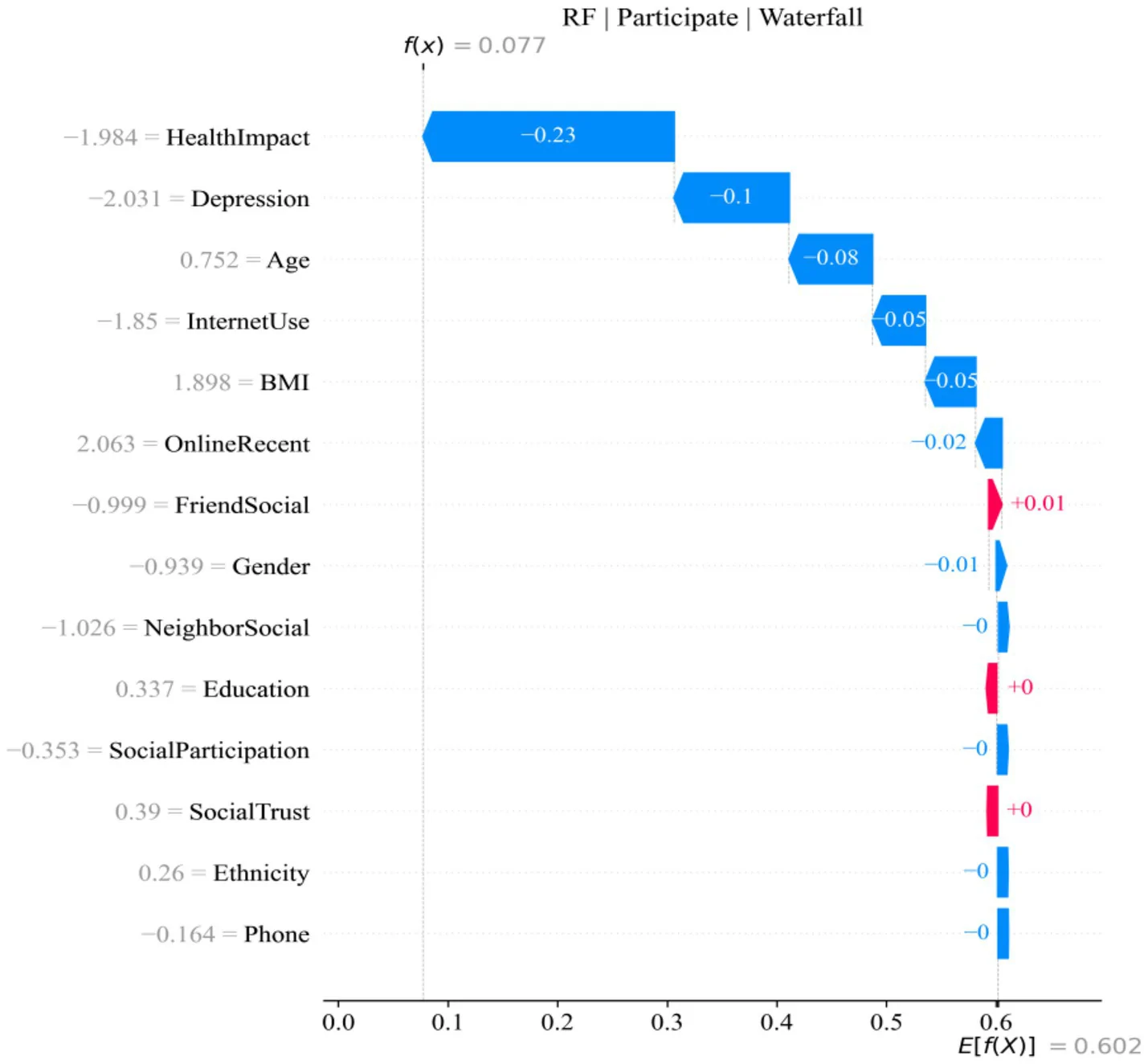
SHAP waterfall plot for an individual prediction in the Participate group.

In the SHAP summary scatter plot for the Non-Participate group (Figure 9), there are marked differences in the direction of contribution of different features to the model’s output. The results show that Health Impact remains the most significant variable influencing the model’s predictions; high Health Impact values correspond primarily to positive SHAP values, whilst low values are more frequently found in the negative SHAP region. Depression also exhibits a strong influence. Age displays a trend opposite to that of Health Impact. Furthermore, ‘Friend Social’ and ‘Neighbour Social’ generally exerted a certain degree of influence, whilst the SHAP values for variables such as ‘Internet Use’ and ‘Education’ were generally close to zero, indicating a relatively limited impact on the model’s output.

**Figure 9:**
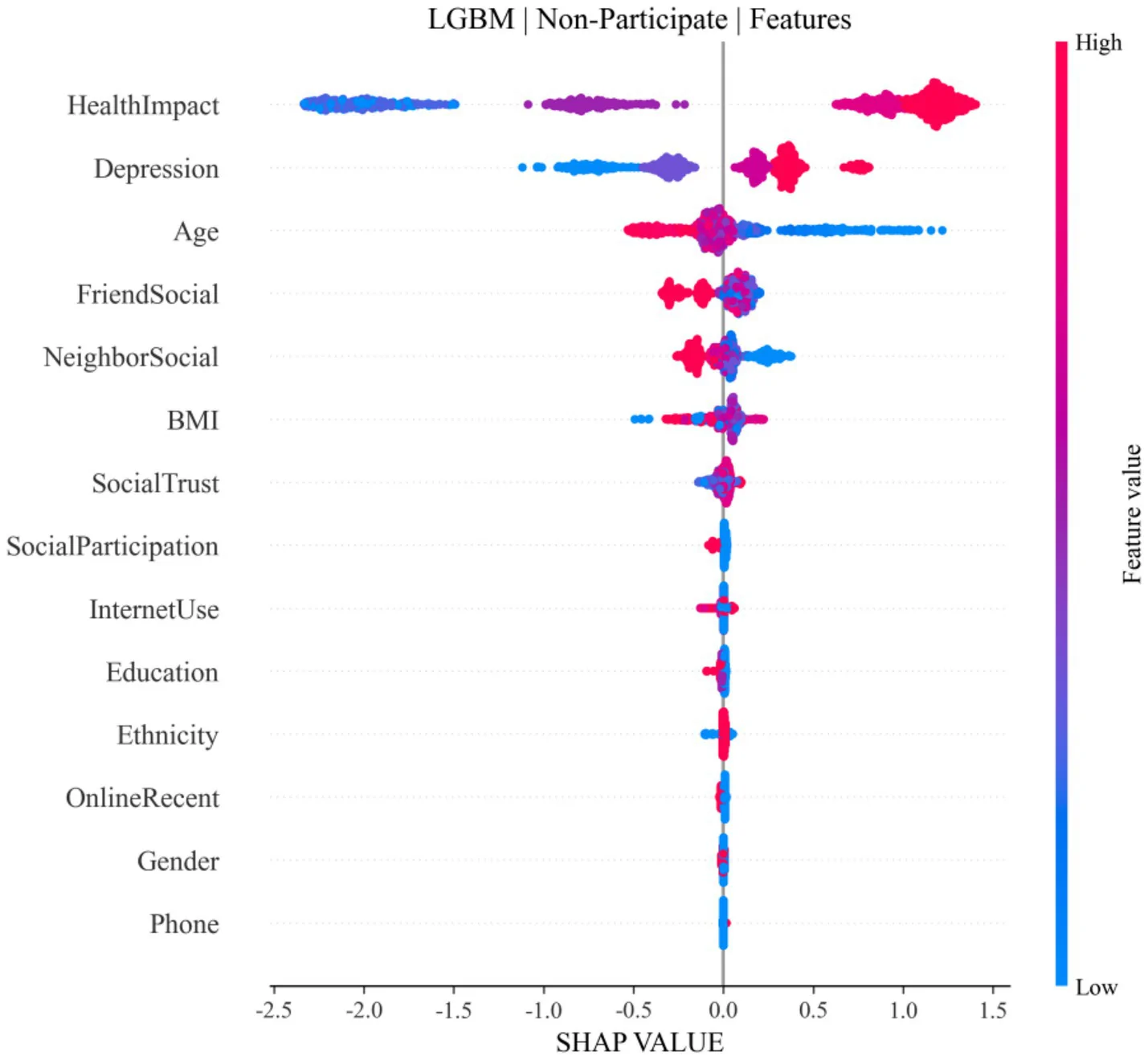
SHAP summary plot of feature contributions in the Non-Participate group.

Figure 10 presents the ranking of the average absolute SHAP values for each feature in the Non-Participate group. ‘Health Impact’ remains the most important, followed by ‘Depression’ and ‘Age’. ‘Friend Social’ and ‘Neighbour Social’ rank in the second tier in terms of importance; BMI possesses some predictive value, whilst the importance of variables such as ‘Social Trust’ and ‘Social Participation’ is relatively low, contributing little to the model’s predictions. Overall, model predictions in the Non-Participate group are primarily driven by a small number of key variables, with ‘Health Impact’ being significantly more important than the others.

**Figure 10:**
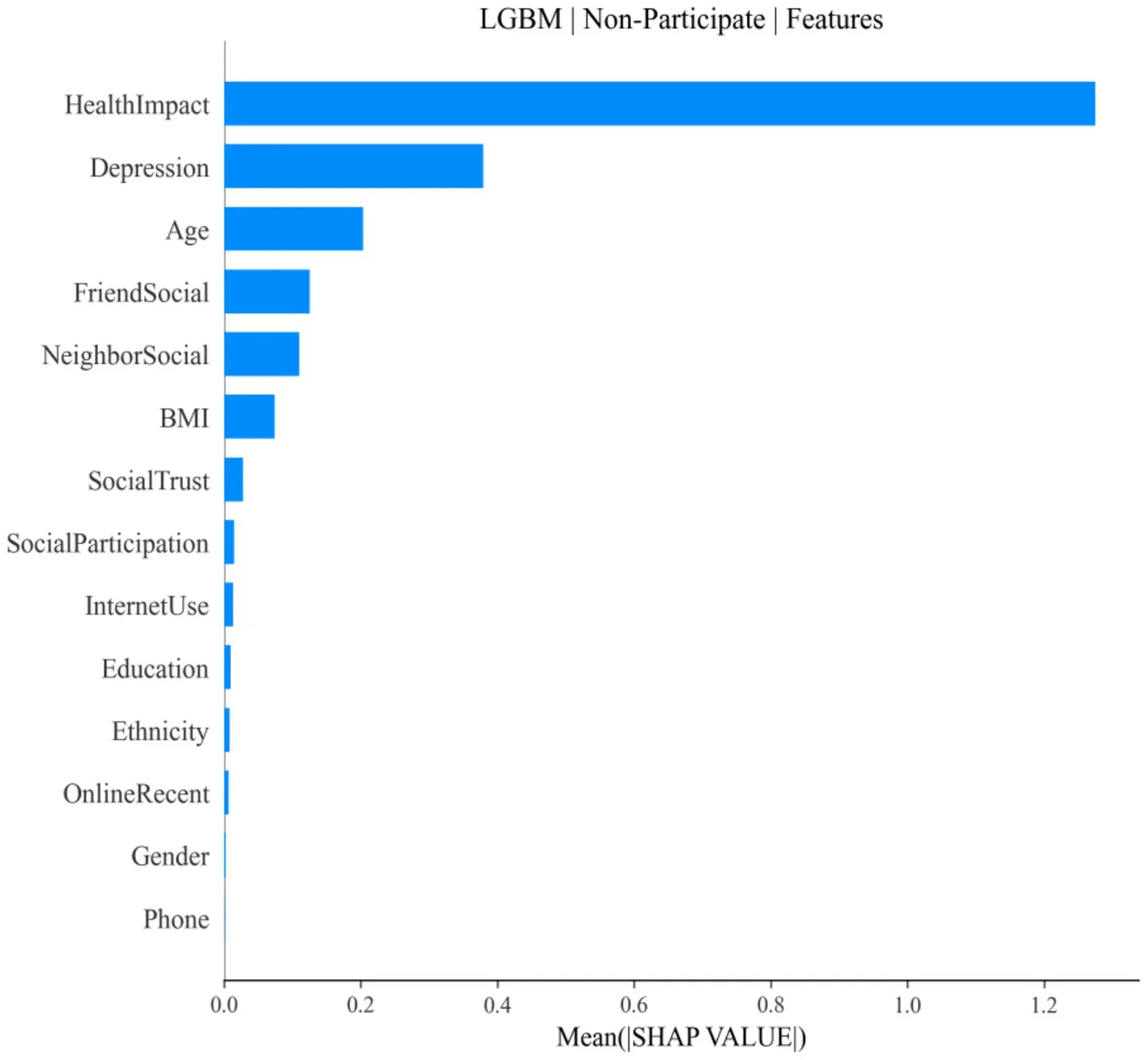
Mean absolute SHAP value importance ranking in the Non-Participate group.

Figure 11 displays the individual feature contribution explanation plot for a single sample in the Non-Participate group. The SHAP model output for this sample is −4.047, representing a further reduction compared to the model’s baseline value of E(f(X)) = −0.516. Among these, Health Impact makes the largest negative contribution to the prediction result, followed by Depression and Age; these three variables collectively constitute the primary influencing factors in the model’s prediction. Education, Social Trust and BMI also contribute to a certain extent in a negative manner.

**Figure 11:**
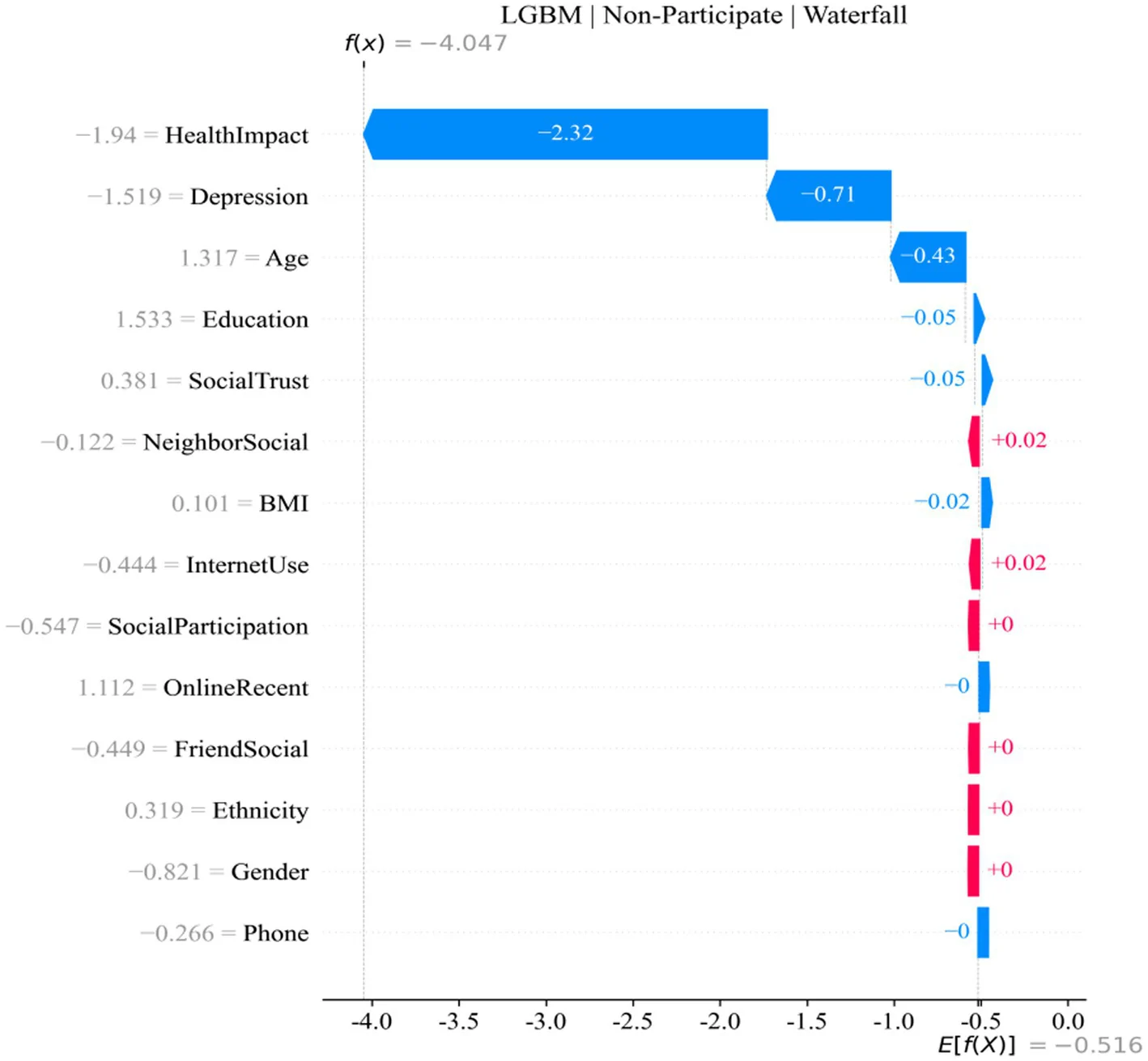
SHAP individual feature contribution explanation plot in the Non-Participate group.

### 3.5 Sensitivity analyses

The physical exercise participation group was further subdivided according to exercise frequency into a Regular participation (those who exercised daily or several times a week) and an Occasional participation (those who exercised several times a month or several times a year or less’), and seven machine learning models were rebuilt for each group, respectively. The ROC AUC values for the Regular participation on the test set ranged from 0.768 to 0.817, with the RF value being relatively high (0.817, 95% CI: 0.791–0.842), representing an improvement on the RF value of 0.808 (95% CI: 0.786–0.832) observed in the Participate group in the main analysis; In the Occasional participation, the ROC AUC ranged from 0.695 to 0.777, with RF also relatively high (0.777, 95% CI: 0.737–0.816), though overall lower than that of the‘Regular participation group and below the performance of most primary models in the Participate group of the main analysis. SHAP analysis revealed that the core predictors were generally consistent across both groups, with Health Impact, Age and Depression all ranking highly in terms of importance; however, there were some differences in their relative contributions: in the Regular participation, Age was more important than Depression, whilst in the Occasional participation, Depression was more important than Age. The calibration curves for both groups were generally close to the 45° reference line. Overall, following further stratification by exercise frequency, some differences emerged in model performance and the ranking of importance for certain features, although the core predictive factors remained generally stable (see Supplementary Figures 1–8).

To further assess the contribution of Health Impact to the models’ predictive performance, this study conducted an ablation analysis, in which the machine learning models were rebuilt after removing the Health Impact variable. The results showed that the discriminatory power of each model decreased slightly compared with the main analysis, but the ranking of importance for the main predictive features remained generally stable. Following the removal of Health Impact, the ROC AUC for each model decreased compared with the main analysis; however, the models still retained a certain degree of discriminatory power. SHAP results indicated that Depression, Age and Friend Social were the primary predictive features. The calibration curves for each model were generally close to the 45° reference line, as shown in Tables 5, 6.

**Table 5:** Comparison of model performance before and after health impact ablation in the Participate group.

Group	Model	Before 95% CI	After 95% CI
Participate	RF	0.808 (0.786–0.832)	0.758 (0.734–0.783)
	DT	0.787 (0.764–0.810)	0.724 (0.700–0.751)
	XGB	0.807 (0.785–0.831)	0.752 (0.727–0.777)
	LGBM	0.805 (0.782–0.829)	0.755 (0.731–0.781)
	SVM	0.792 (0.769–0.817)	0.750 (0.725–0.775)
	LR	0.801 (0.778–0.825)	0.761 (0.737–0.786)
	KNN	0.744 (0.718–0.770)	0.700 (0.675–0.727)

**Table 6:** Comparison of model performance before and after health impact ablation in the Non-Participate group.

Group	Model	Before 95% CI	After 95% CI
Non Participate	RF	0.804 (0.771–0.833)	0.735 (0.701–0.771)
	DT	0.780 (0.747–0.811)	0.705 (0.669–0.742)
	XGB	0.804 (0.772–0.833)	0.725 (0.689–0.760)
	LGBM	0.806 (0.775–0.835)	0.736 (0.701–0.771)
	SVM	0.805 (0.773–0.834)	0.704 (0.666–0.740)
	LR	0.804 (0.770–0.833)	0.736 (0.700–0.769)
	KNN	0.783 (0.749–0.813)	0.690 (0.654–0.727)

Furthermore, this study selected four representative models—RF, XGBoost, LGBM and LR—to conduct a sensitivity analysis whilst retaining the original five-class classification of SRH outcomes. The results showed that the ROC AUC values for each model in the Participate Group ranged from 0.769 to 0.786, with RF yielding a relatively high value (0.786), followed by XGBoost (0.781), LR (0.779) and LGBM (0.769); in the non-participation group, they ranged from 0.761 to 0.777, with LGBM performing relatively best (0.777), followed by XGBoost (0.776), RF (0.773) and LR (0.761). Compared with the main binary classification analysis, the AUC of the five-classification models showed a slight overall decline; however, the relative performance patterns of the models across the two groups were generally similar. The accuracy of the five-class models ranged from 0.42 to 0.48, which was lower than that observed in the binary classification analysis. Overall, after retaining the original five-class SRH information, the models still demonstrated a certain degree of discriminatory power, and the relative performance patterns across models were generally consistent with those of the main binary classification analysis; detailed results are presented in Supplementary Tables 1, 2.

## 4 Discussion

Based on the CGSS 2021 data, this study categorized adults into a Participate group and a Non-Participate group according to their physical activity status. Seven machine learning models were developed to predict their SRH status, and the SHAP method was used to identify key predictors and their relative contributions within each group. The results showed that, compared with the Non-Participate group, the Participate group had a higher proportion of participants reporting good SRH and exhibited certain differences in demographic, health status, and social behavior characteristics. Model comparison results indicate that RF exhibited a relatively high ROC AUC 0.808 (0.786–0.832) in the Participate group, while LGBM exhibited a relatively high ROC AUC 0.806 (0.775–0.835) in the Non-Participate group. However, the performance differences between the leading models in the two groups were generally small. Further SHAP analysis revealed that Health Impact, Age, and Depression were common core predictors across both groups, though their order of importance and distribution of contributions were not entirely consistent. Variables such as Internet Use, Education, Social Friends, and BMI also demonstrated some predictive value, suggesting that, in addition to health and psychological factors, educational background, digital behaviors, and social engagement also provide some information for adults to assess their own health prospects. Furthermore, after conducting sensitivity analyses at different levels—including variations in physical activity grouping methods—the core predictive factors and their overall patterns of importance remained largely consistent, although model performance and the ranking of some features varied across different analytical scenarios. Overall, the prediction of SRH among adults under different physical activity participation statuses exhibits both commonalities and certain differences; the findings of this study may provide a basis for understanding the predictive structure of SRH among different groups of physically active individuals, and serve as a reference for conducting subsequent stratified health studies and implementing tailored health promotion programs.

In this study, the RF, LGBM, and XGBoost models demonstrated relatively stable overall performance, suggesting that ensemble learning models capable of handling nonlinear relationships and variable interactions are well-suited for predicting SRH. The performance differences among the models were minor, suggesting that predictive ability likely stems from the health information contained in the input variables rather than the absolute superiority of any single algorithm. Therefore, identifying stable and interpretable predictive factors may hold greater practical value than focusing on minor differences in model performance. The results of this study not only further validate the application potential of machine learning in health prediction but also reflect the multifactorial, nonlinear nature, and population heterogeneity of SRH prediction among adults.

To further understand the contribution characteristics of predictors of SRH across different physical activity patterns, this study employed the SHAP method to conduct an interpretability analysis of the models. The results showed that Health Impact, Age, and Depression were the most important predictors across all Participate groups, indicating that physical health status, age, and recent emotional state form the critical foundation for adults’ SRH—a finding consistent with previous research (55). It is worth noting that the relative importance of Age and Depression varies across different physical activity groups, suggesting that the relative contribution of these health-related factors to model predictions may be heterogeneous depending on the level of physical activity participation.

Age not only reflects changes in physiological function, the accumulation of chronic diseases, and alterations in daily activity capacity but is also closely related to sociodemographic characteristics and health-related factors; therefore, it continuously influences an individual’s assessment of their overall health status (56, 57). In contrast, recent feelings of depression or low mood more accurately reflect an individual’s current psychological state and influence their evaluation of their own health status. Previous studies have shown that physical exercise is closely associated with both physical function and emotional state, and its effects are influenced by factors such as exercise frequency, intensity, duration, and an individual’s baseline health status (58–60). Therefore, the differences in the relative contributions of Age and Depression between the Participate group and the Non-Participate group in this study suggest that physical exercise participation may have altered the relative roles of age-related health burden and recent emotional state in the formation of SRH.

This study further analyzed exercise participants by exercise frequency to examine the heterogeneity of predictive factors across different exercise frequencies. The results showed that the key predictive variables remained generally consistent across groups, indicating that physical health status, age, and recent emotional state remain core predictors of SRH; however, the model predictive performance was higher in the regular participation group than in the overall Participate group, while the occasional Participate group exhibited relatively lower predictive performance, it is noted that there are some differences in the predictive structures and model performance across groups with different exercise frequencies. Previous research suggests that regular physical exercise is a crucial component of stable health behavior patterns; individuals who maintain long-term exercise habits typically exhibit more consistent lifestyles, more stable physical functioning, and more stable psychological states, resulting in a more stable correlation between health-related characteristics and SRH (61). By contrast, occasional exercisers may exhibit greater individual variation in terms of exercise regularity, health behaviors, baseline health status and recent emotional experiences, which may increase the complexity of the relationship between health-related characteristics and SRH in this group (62, 63). Therefore, incorporating exercise frequency into the analysis further reveals differences in the contribution characteristics of predictors of SRH across different exercise frequencies.

“Health Impact”—that is, the extent to which health issues affect daily activities—exhibited a high predictive contribution in the Participate group, the Non-Participate group, and subgroups with different exercise frequencies. This variable reflects the impact of health status on daily activities such as work, housework, and social participation; it can relatively directly characterize an.

individual’s functional limitations and health burden in real life, and may comprehensively encompass multidimensional information such as limitations in physiological function, psychological state, and social roles (64). Previous studies have shown that limitations in daily functioning are closely associated with SRH. Even after controlling for the number and types of chronic diseases, the activity limitations actually experienced by individuals may still significantly influence their assessment of their overall health status (65, 66). Large-scale population studies have also found that, compared to disease indicators alone, activity limitations in real life may more directly reflect an individual’s subjective experience of health (55). Furthermore, limitations in daily activities caused by health problems often co-occur with psychological issues such as depression and low mood; the combined effect of physical functional limitations and psychological burden may further strengthen the association between Health Impact and SRH (67).

It should be noted that Health Impact and SRH share some conceptual and measurement overlap. The former measures the specific impact of health problems on work or daily activities, while the latter is an individual’s general assessment of their overall health status; both involve subjective perceptions of one’s health status but are not entirely identical. To assess whether the model over-relies on Health Impact, this study further conducted an ablation analysis by removing this variable to compare changes in model performance and the structure of feature importance. The results showed that after removing Health Impact, the discriminatory power of both the Participate group and Non-Participate group models decreased somewhat, but they still retained a certain level of predictive performance, and variables such as Age and Depression continued to rank among the most important features. This indicates that while “Health Impact” does indeed contain strong information related to SRH and serves as an important predictive feature for the model, the model’s predictive structure is not driven solely by this variable; demographic, psychological, and social behavior characteristics continue to provide supplementary predictive information. Given that this study utilized cross-sectional data, the above results reflect predictive contributions and association structures; the causal relationship between “Health Impact” and SRH should be interpreted with caution.

Following the ablation analysis, “Friend Social” and “Internet Use” rose in the feature importance ranking, indicating that, when Health Impact was excluded, the relative contributions of social interaction and digital behavior variables in the model increased. Social interaction with friends can influence an individual’s perception of health by providing emotional support, alleviating loneliness, and promoting cognitive stimulation and participation in daily activities (68). Broader social participation, such as community activities and interest groups, may also have a positive impact on SRH by enhancing a sense of belonging, life satisfaction, and support from social resources (69). Internet use can expand access to health information and social connections and help individuals maintain remote interactions, thereby influencing psychological well-being and health evaluations (70, 71). Relevant systematic reviews also indicate that moderate use of the Internet and social media may strengthen social connections and alleviate social isolation (72). However, it should be noted that an increase in feature ranking reflects relative predictive contributions within the model and does not equate to causal effects or completely independent roles.

In addition to the core features, variables such as Education and BMI also play a supplementary role in predicting SRH. A higher level of education may influence SRH by improving health literacy, adherence to healthy behaviors, and self-assessment abilities (73, 74); BMI, on the other hand, reflects an individual’s physiological status, and values that are too high or too low are typically associated with poorer SRH (75). Although the predictive contributions of these variables are relatively low, they help the model identify an individual’s health status more comprehensively across dimensions such as social factors, behavior, and access to resources. Therefore, future stratified health research and health promotion initiatives could, whilst focusing on physical and mental health, further take into account factors such as educational background, social interaction and the use of digital health resources, whilst also examining differences in the relative contributions of these factors amongst different groups of people who engage in physical activity.

A key strength of this study is the use of physical activity participation status as a stratification dimension. This enabled the separate development of SRH prediction models for Participants and Non-participants and the use of SHAP to compare the relative contributions of key features, thereby facilitating a direct comparison of SRH prediction structures across groups with different physical activity participation status. Using CGSS 2021 data, this study incorporated multidimensional characteristics—including demographic, health and psychological, information usage, and social behavior—and compared the predictive performance of seven machine learning models. Sensitivity analyses produced findings that were generally consistent with the primary predictive features, providing supplementary evidence for the robustness of the main findings. The results indicate that Health Impact, Age, and Depression demonstrated high predictive importance across all levels of physical exercise participation, whereas variables such as Education, BMI provided supplementary predictive information; the relative contributions of certain features differed between groups. These findings may provide a reference for subsequent studies seeking to further validate the predictive structure of SRH among different groups of physical exercise participants, conduct stratified health research, and explore differentiated health promotion strategies. Given that this study is a cross-sectional analysis of secondary data, these findings should be interpreted with caution.

## 5 Limitations

This study has several limitations. First, the data were derived from a cross-sectional survey, which limits inferences regarding causal relationships. Second, SRH and information on certain health behaviors, social behaviors, and physical activity were based on respondents’ self-reports and may therefore be subject to recall and social desirability biases. Third, both the model development and evaluation in this study were based on CGSS 2021 data. Although the sensitivity analyses suggested that the core predictive structure was relatively robust, its generalizability to other survey years, regions, and populations remains to be externally validated. Furthermore, as the model development and evaluation in this study were based on an analytical sample that did not utilize survey sampling weights, the model’s performance and the importance of its features primarily reflect the predictive structure within the current analytical sample. Under complex sampling designs, there are differences in the representativeness of different individuals within the population, which may have some impact on model performance, predictive relationships and the ranking of variable importance; therefore, the applicability of the study’s findings at the national population level should be interpreted with caution. Fourth, although this study quantified the predictive contributions of core and secondary predictors, it did not examine interactions among predictors or potential heterogeneity in their effects across population subgroups.

Future research could employ longitudinal studies, combining other objective physical activity measurement methods to examine the temporal associations between physical exercise, recent emotional states, and other core predictors with SRH. At the same time, external validation will be carried out across different years, regions and population samples, and weighted and unweighted models will be compared in order to assess the stability and overall applicability of the model’s predictions. Building on this, it would also be possible to analyze the interactions between core and secondary predictors and their variations across different populations, and to further explore the potential roles of social and digital behaviors in individual health assessment and health promotion, thereby providing a basis for the development of more targeted public health strategies.

## 6 Conclusion

Based on the CGSS 2021 data, this study employed machine learning models and the SHAP method to compare the predictive performance and key predictors of SRH among adults with different levels of physical activity participation. The findings revealed that Health Impact, Age, and Depression were relatively stable core predictors across different Participate groups, while variables such as educational level, internet use, social interaction, and BMI provided supplementary predictive information. While there were some differences in model performance and the relative contributions of certain features among groups with different levels of physical activity participation and exercise frequency, the overall predictive structure showed good consistency. This study contributes to an understanding of the commonalities and differences in the predictive structure of SRH across different levels of physical activity participation, and provides a reference for future stratified health research and tailored health promotion. Given that this study is based on data from a single cross-sectional survey, the model requires external validation in independent cohorts, in surveys conducted in other years, or in samples from different geographical regions; its practical value must therefore be assessed with caution, subject to further validation.

## Data Availability

The original contributions presented in the study are included in the article/Supplementary material, further inquiries can be directed to the corresponding author.
