# Supplementary material for: Machine learning prediction of self-rated health among adults under different physical exercise participation statuses
Source: Front Public Health. 2026 Oct 9;14:1882082. doi: 10.3389/fpubh.2026.1882082 (PMC13690745; doi:10.3389/fpubh.2026.1882082)
Supplement: Supplementary file 1 [file Data_Sheet_1.PDF]

Supplementary Material

1 Supplementary Figures and Tables

1.1 Supplementary Figures

1.1.1 Exercise_sensitivity

Supplementary Figure 1. ROC curves for the occasional participation.

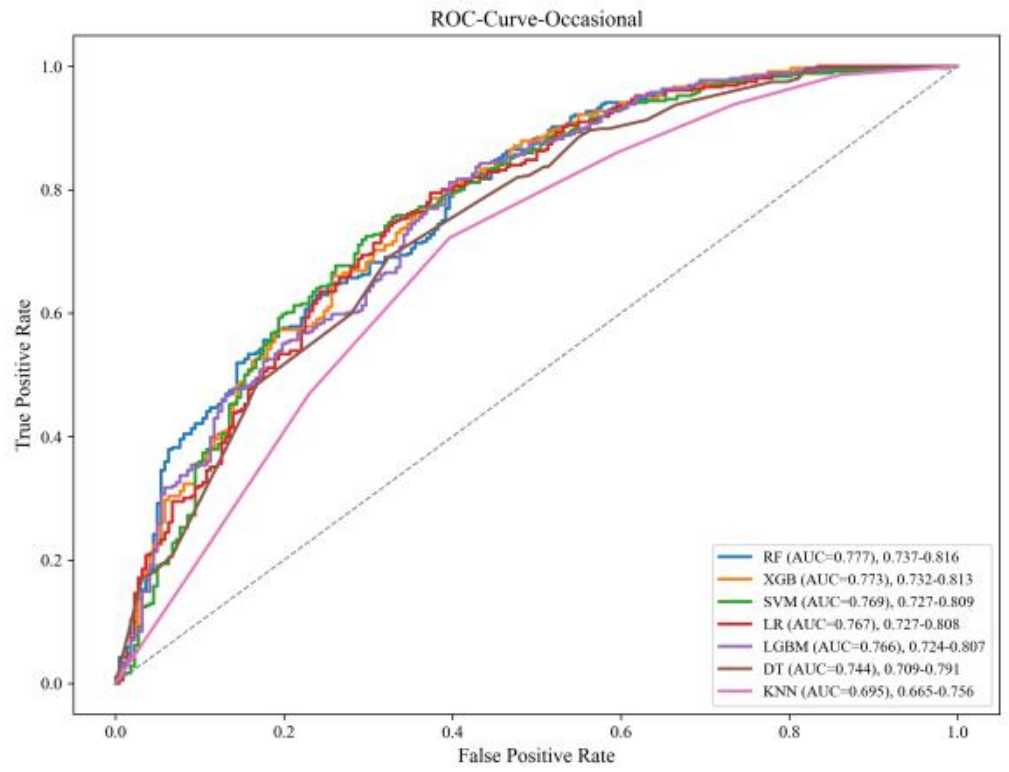

Supplementary Figure 2. Calibration curves for the occasional participation.

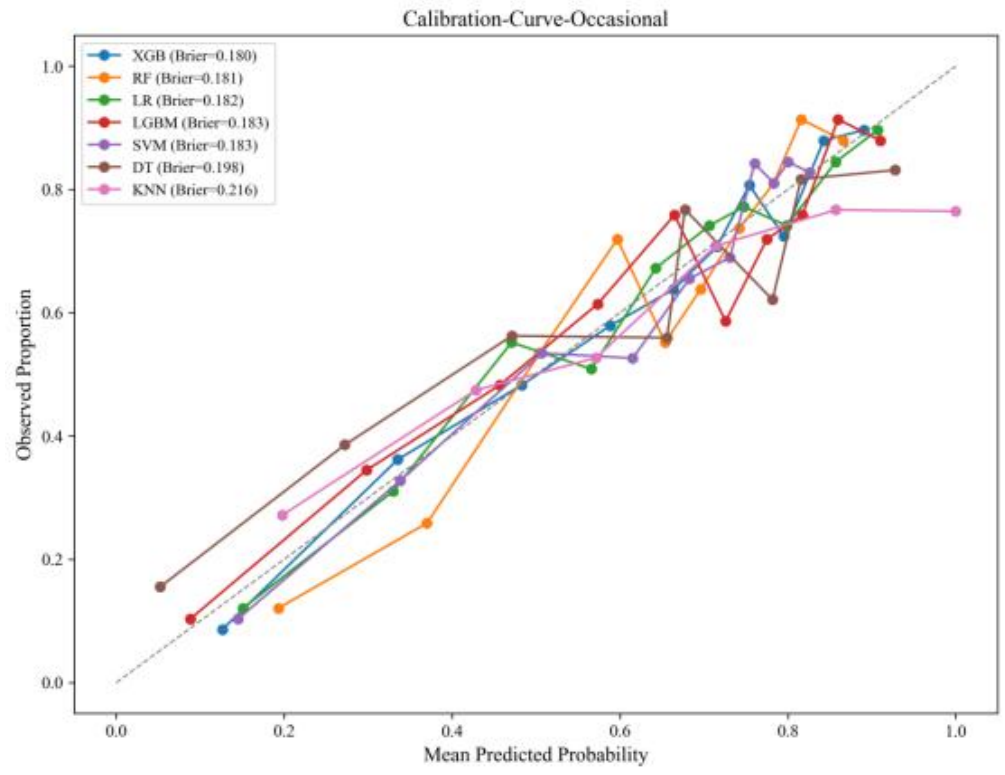

Supplementary Figure 3. ROC curves for the regular participation.

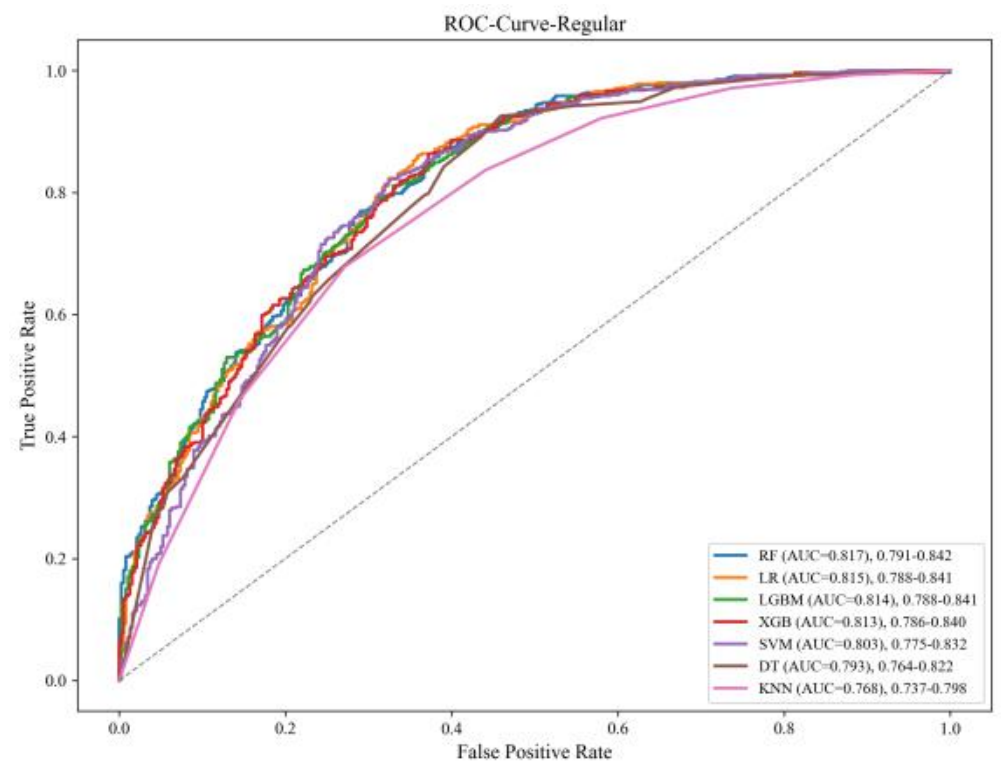

Supplementary Figure 4. Calibration curves for the regular participation.

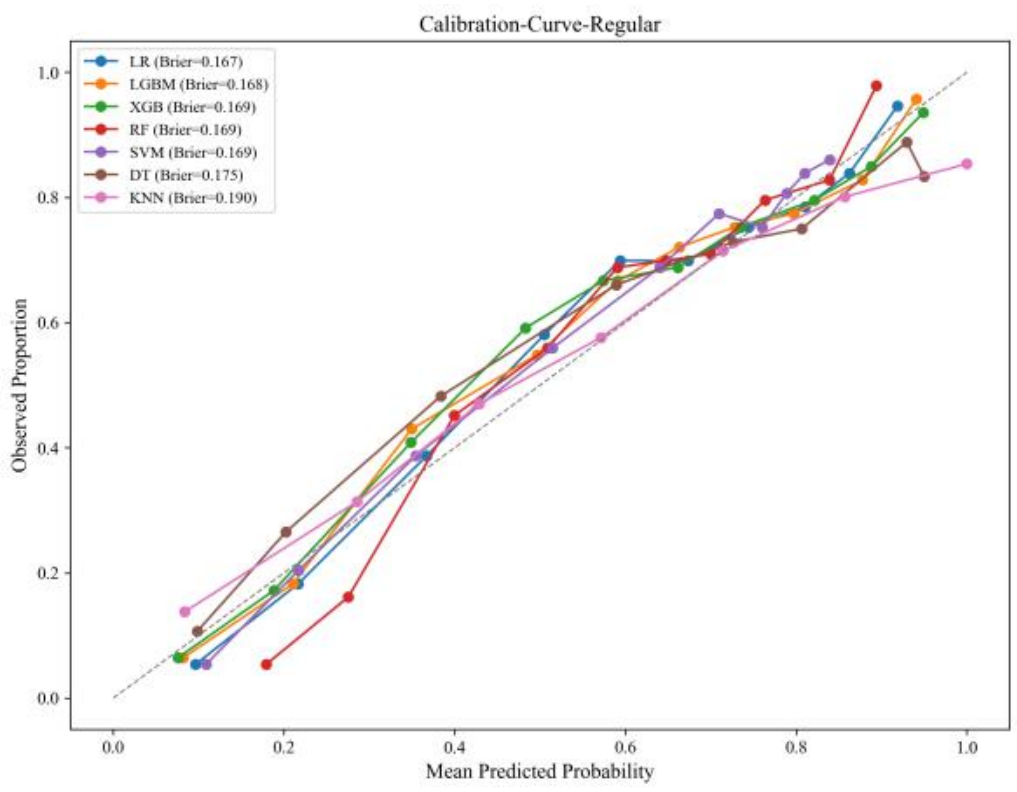

Supplementary Figure 5. SHAP summary plot for the occasional participation.

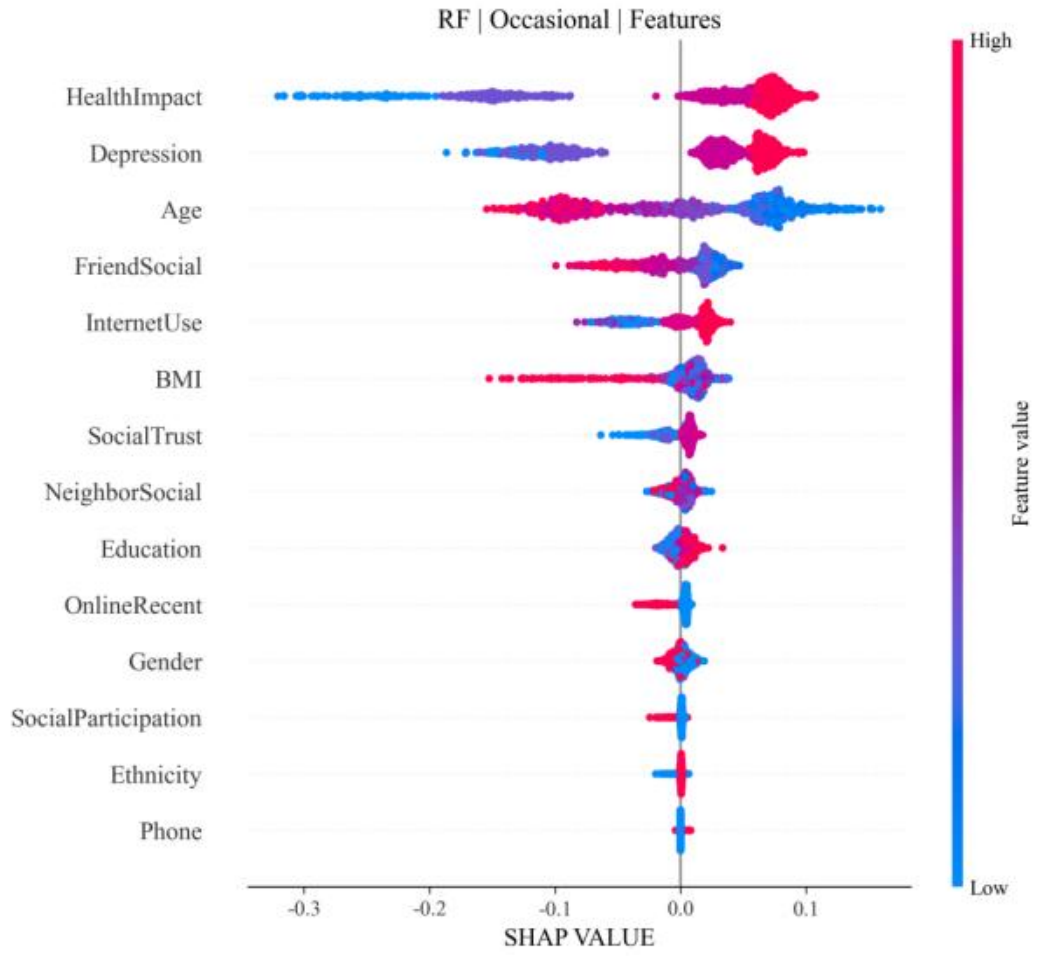

Supplementary Figure 6. SHAP importance plot for the occasional participation.

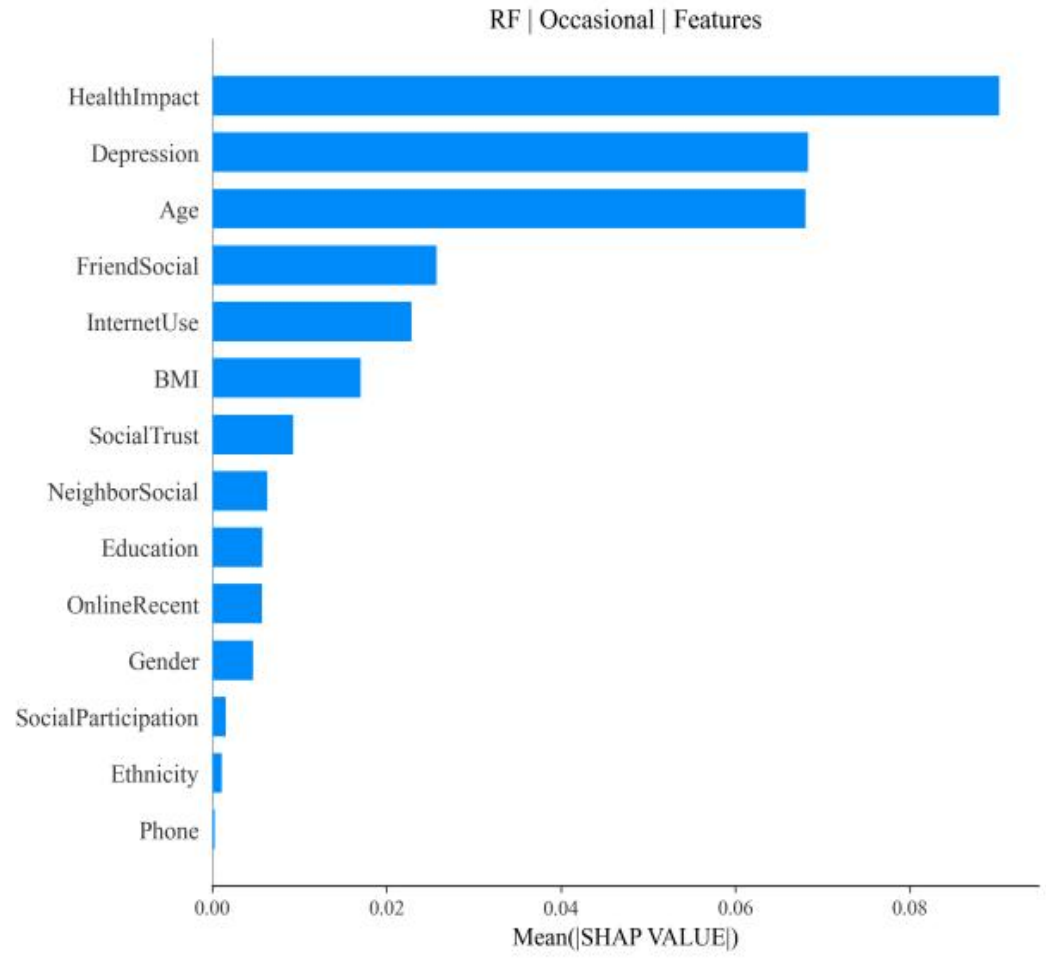

Supplementary Figure 7. SHAP summary plot for the regular participation.

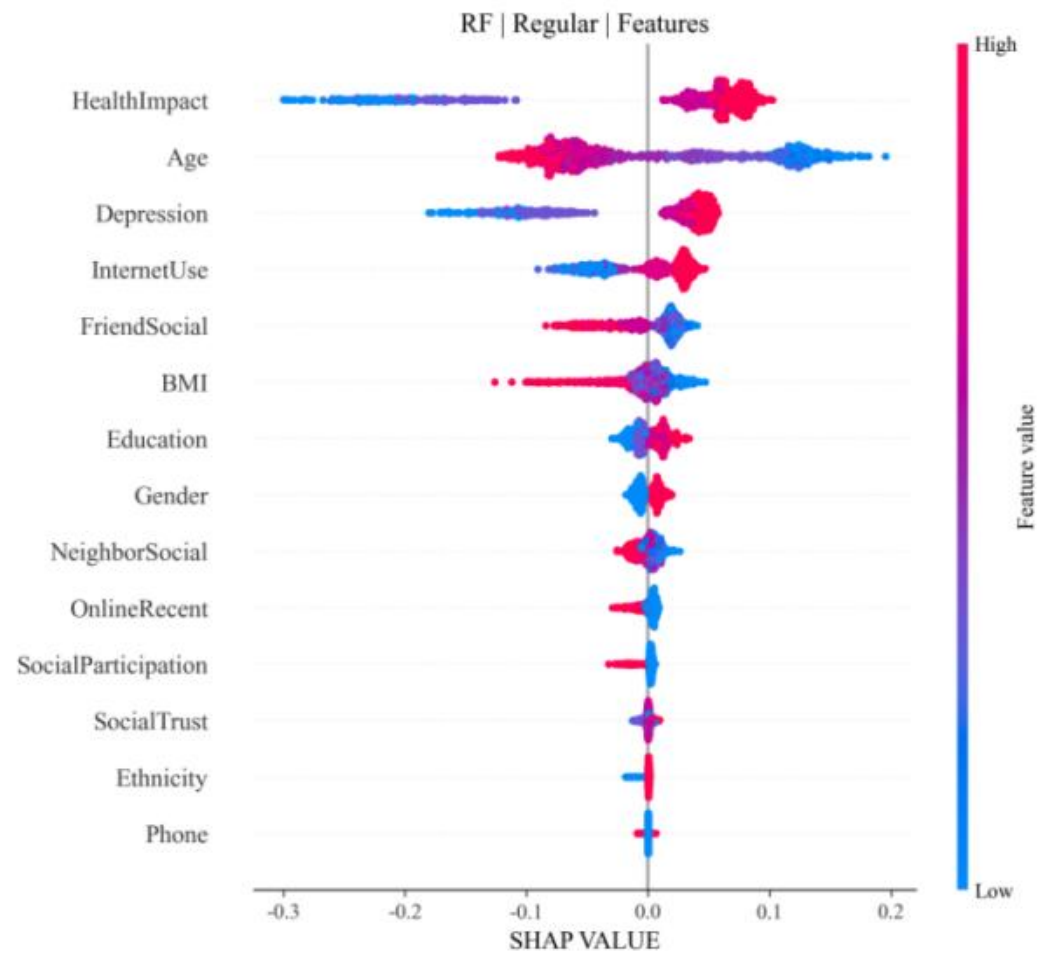

Supplementary Figure 8. SHAP importance plot for the regular participation.

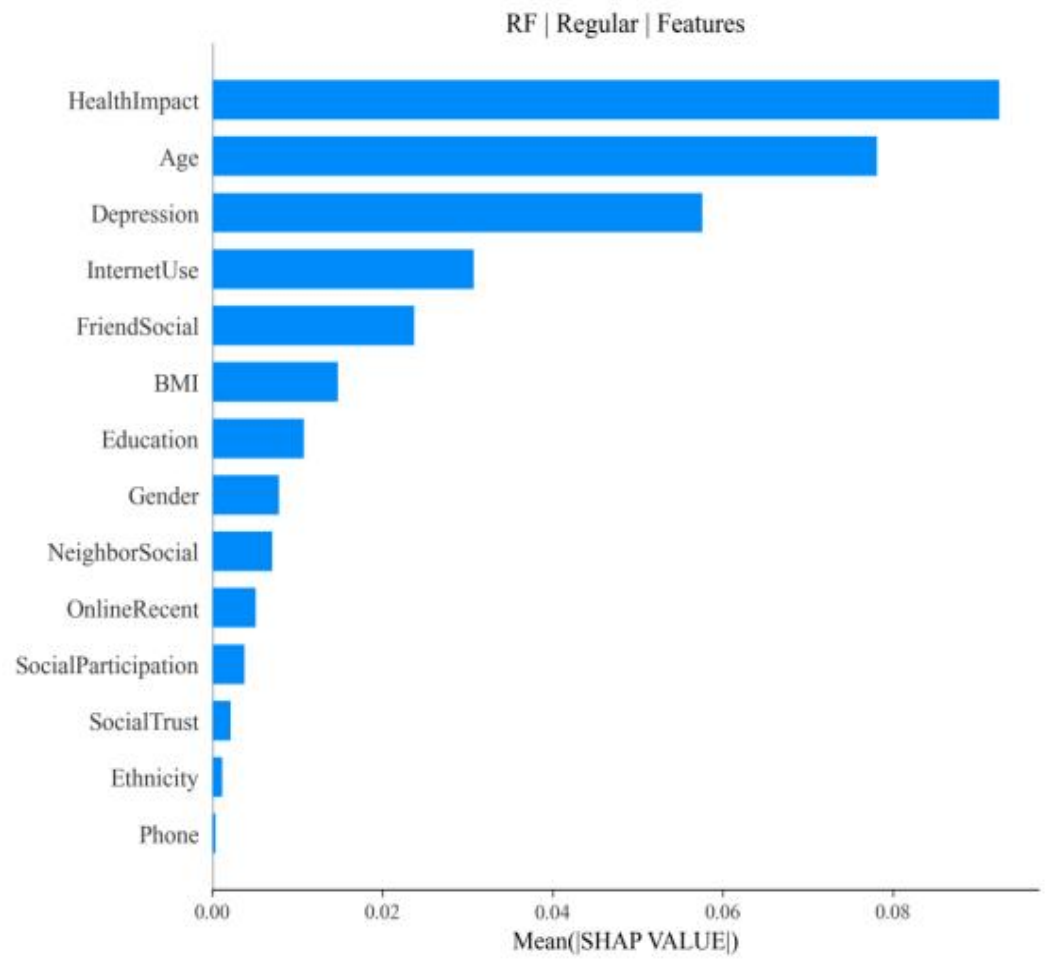

1.2 Supplementary Table

1.2.1 SRH Sensitivity Analysis

Table 1 Optimal hyperparameter configurations for the Participate group

Group	Model	Test Accuracy	Test Balanced Accuracy	Test Macro F1	Test Weighted F1	Test Macro ROC AUC
**Participate**	RF	0.481	0.353	0.375	0.447	0.786
	XGB	0.472	0.384	0.410	0.457	0.781
	LGBM	0.471	0.391	0.413	0.459	0.769
	LR	0.479	0.368	0.391	0.460	0.779

Table 2 Optimal hyperparameter configurations for the Non-Participate group

Group	Model	Test Accuracy	Test Balanced Accuracy	Test Macro F1	Test Weighted F1	Test Macro ROC AUC
**Non Participate**	RF	0.451	0.384	0.376	0.412	0.773
	XGB	0.453	0.415	0.422	0.439	0.776
	LGBM	0.443	0.404	0.410	0.427	0.777
	LR	0.422	0.391	0.387	0.399	0.761

1.2.2 Descriptive Statistics

Table3 Descriptive characteristics of participants by physical exercise participation status

Feature variable	Total (%) (N = 7,529)	Participate group(%) (N = 5,025, 66.7%)	Non-participate group(%) (N = 2,504, 33.3%)	χ^2/t	P
Age	50.24 (16.71)	47.35 (17.02)	56.03 (14.42)	23.166	<0.001
BMI	23.09 (3.69)	23.04 (3.59)	23.17 (3.88)	1.367	0.172
Health impact	3.98 (1.21)	4.14 (1.08)	3.66 (1.38)	15.232	<0.001
Depression	3.95 (1.08)	4.03 (1.01)	3.81 (1.19)	8.059	<0.001
Internet use	3.43 (1.64)	3.77 (1.50)	2.76 (1.69)	25.363	<0.001
Social trust	3.63 (1.00)	3.63 (0.96)	3.61 (1.06)	0.899	0.369
Neighbor social	4.27 (2.22)	4.26 (2.18)	4.28 (2.29)	0.298	0.765
Friend social	4.17 (1.95)	3.83 (1.82)	4.86 (2.03)	21.467	<0.001
**Gender**				18.603	<0.001
Female	4120 (54.7)	2662 (53.0)	1458 (58.2)		
Male	3409 (45.3)	2363 (47.0)	1046 (41.8)		
**Ethnicity**				5.075	0.024
Other ethnic minorities	556 (7.4)	347 (6.9)	209 (8.3)		
Han Chinese	6973 (92.6)	4678 (93.1)	2295 (91.7)		
**Education**				1096.93	<0.001
Illiterate/primary school or below	2298 (30.5)	1035 (20.6)	1263 (50.4)		
Junior high school	2197 (29.2)	1367 (27.2)	830 (33.1)		
Senior high/technical secondary/vocational school or equivalent	1414 (18.8)	1125 (22.4)	289 (11.5)		

College or above	1506 (20.0)	1387 (27.6)	119 (4.8)		
Postgraduate or above	114 (1.5)	111 (2.2)	3 (0.1)		
**Phone**				85.127	<0.001
Yes	7228 (96.0)	4898 (97.5)	2330 (93.1)		
No	301 (4.0)	127 (2.5)	174 (6.9)		
**Online Recent**				539.9	<0.001
Yes	5473 (72.7)	4076 (81.1)	1397 (55.8)		
No	2056 (27.3)	949 (18.9)	1107 (44.2)		
**Social participation**				180.506	<0.001
Participated	6394 (84.9)	4464 (88.8)	1930 (77.1)		
Did not participate	1135 (15.1)	561 (11.2)	574 (22.9)		
**SRH**				176.405	<0.001
Unhealthy group (fair, relatively unhealthy, or very unhealthy)	3403(45.2)	2001 (39.8)	1402 (56.0)		
Healthy group (relatively healthy or very healthy)	4126 (54.8)	3024 (60.2)	1102 (44.0)		

Note: Continuous variables are presented as mean(standard deviation, SD), and categorical variables are presented as n (%). Percentages in subgroup columns are calculated within each subgroup. For the χ^2/t statistic column, t tests were used for continuous variables and χ^2 tests were used for categorical variables. P values less than 0.001 are reported as <0.001.

1.2.3 Variable Encoding

Table 4.Definitions, Coding, and Questionnaire Descriptions of Variables Used in the Study

Variable	Data type	Coding/ scale	Original questionnaire item/description
**Age**	Continuous variable	Numeric	Actual age, in years
**BMI**	Continuous variable	Numeric	Calculated as weight (kg) / height (m^2)
**Health Impact** (Frequency of health problems affecting work or daily activities in the past 4 weeks）	Ordinal variable	1–5	1 = Always; 2 = Often; 3 = Sometimes; 4 = Rarely; 5 = Never
**Depression** (Frequency of feeling depressed or upset in the past 4 weeks)	Ordinal variable	1–5	1 = Always; 2 = Often; 3 = Sometimes; 4 = Rarely; 5 = Never
**Internet Use**（Frequency of Internet Use in the Past Year）	Ordinal variable	1-5	1 = Never; 2 = Rarely; 3=Sometimes; 4=Often; 5 = Very frequently
**Social trust**	Ordinal variable	1–5	1 = Strongly disagree; 2 = Disagree; 3 = Neither agree nor disagree; 4 = Agree; 5 = Strongly agree
**Neighbor Social** (Frequency of social/recreational activities with neighbors)	Ordinal variable	1–7	1 = Almost daily; 2 = 1–2 times/week; 3 = Several times/month; 4 = About monthly; 5 = Several times/year; 6 = Once/year or less; 7 = Never
**Friend Social** (Frequency of social/recreational activities with friends)	Ordinal variable	1–7	1 = Almost daily; 2 = 1–2 times/week; 3 = Several times/month; 4 = About monthly; 5 = Several times/year; 6 = Once/year or less; 7 = Never
**Gender**	Categorical variable	0	Female
		1	Male
**Ethnicity**	Categorical variable	0	Other ethnic minority groups
		1	Han ethnicity
**Education** (Highest educational attainment)	Ordinal variable	1	Illiterate / primary school or below
		2	Junior high school
		3	Senior high school / secondary specialized school / vocational high school / technical school or equivalent
		4	College or above
		5	Postgraduate degree or above

**Phone** (Ownership of a personal mobile phone)	Categorical variable	1	Yes
		2	No
**Online Recent** (Internet use in the past six months)	Categorical variable	1	Yes
		2	No
**Social Participation** (Participation in social visits or socializing during leisure time)	Categorical variable	1	Yes
		2	No
**SRH** (Self-rated health status)	Categorical variable	0	Unhealthy group (very unhealthy、relatively unhealthy、fair) :1-3
		1	Healthy group (relatively healthy、very healthy) :4-5
**Exercise**（Physical Activity Participation）	Categorical variable	0	Non-participation in physical activity (never):5
		1	Participation in physical activity (daily, several times a week, several times a month, or several times a year or less'):1-4

1.2.4 Missing Data

Table 5. Comparison of Characteristics Between Included and Excluded Participants and Missing Data Summary

Variable	Category/Level	Included	Excluded	χ²/t	P value	Effect Size
Age	Mean (SD)	50.24 (16.71)	57.08 (16.94)	-7.450	<0.001	-0.410
BMI	Mean (SD)	23.09 (3.69)	23.01 (3.89)	0.277	0.782	0.021
Health Impact	Mean (SD)	3.98 (1.21)	3.68 (1.35)	3.901	<0.001	0.241
Depression	Mean (SD)	3.95 (1.08)	3.78 (1.23)	2.481	0.014	0.158
Gender	Female	4,120 (54.7%)	218 (61.4%)	6.125	0.013	0.028
	Male	3,409 (45.3%)	137 (38.6%)			
Education	Illiterate/primary school or below	2,298 (30.5%)	189 (56.1%)	101.757	<0.001	0.114
	Junior high school	2,197 (29.2%)	74 (22.0%)			
	Senior high/technical secondary/vocational school or equivalent	1,414 (18.8%)	42 (12.5%)			
	College or above	1,506 (20.0%)	29 (8.6%)			
	Postgraduate or above	114 (1.5%)	3 (0.9%)			
SRH	Unhealthy group (fair, relatively unhealthy, or very unhealthy)	3,403 (45.2%)	201 (57.6%)	20.645	<0.001	0.051
	Healthy group (relatively healthy or very healthy)	4,126 (54.8%)	148 (42.4%)			
Exercise participation	Participate	5,025 (66.7%)	154 (45.0%)	68.533	<0.001	0.093
	Non-participate	2,504 (33.3%)	188 (55.0%)			

Note: Excluded participants refer to age-eligible respondents who were excluded from the complete-case analysis because of missing data. Missing data were observed for BMI (n = 164, 2.08%), Education (n = 18, 0.23%), Health Impact (n = 20, 0.25%), Depression (n = 28, 0.36%), Internet Use (n = 6, 0.08%), Phone (n = 2, 0.03%), Online Recent (n = 9, 0.11%), Social Participation (n = 4, 0.05%), Neighbor Social (n = 29, 0.37%), Friend Social (n = 38, 0.48%), Social Trust (n = 62, 0.79%), SRH (n = 6, 0.08%), and Exercise (n = 13, 0.16%). No missing data were observed for Age, Gender, or Ethnicity.
